# Chaperonin in health and disease

**DOI:** 10.1186/s43556-026-00437-0

**Published:** 2026-04-01

**Authors:** Pengfei Xu, Meijun Ji, Wenhan Zhuang, Yumin Guo, Xiaoge Geng, Jingya Wang, Jiyong Jing, Wensheng Pan, Chenjing Zhang

**Affiliations:** 1 Hangzhou Normal University, Hangzhou, Zhejiang China; 2 Hangzhou Medical College, Hangzhou, Zhejiang China; 3 Cancer Center, Department of Gastroenterology, Zhejiang Provincial People’s Hospital, Affiliated People’s Hospital, Hangzhou Medical College, Hangzhou, Zhejiang China; 4 Institute of Gastrointestinal Diseases, Hangzhou Medical College, Hangzhou, Zhejiang China; 5 Zhejiang Provincial Engineering Laboratory of Diagnosis, Treatment and Pharmaceutical Development of Gastrointestinal Tract Tumors, Hangzhou, Zhejiang China

**Keywords:** Heat shock protein, Chaperonins, HSP60, TRiC/CCT, Disease

## Abstract

Chaperonins, evolutionarily conserved heat shock proteins characterized by subunits of approximately 60 kDa, play indispensable roles in maintaining cellular homeostasis. In eukaryotes, chaperonins include primarily HSP60 and TRiC, with HSP60 being a crucial mitochondrial chaperonin and TRiC being an essential cytosolic chaperonin. The two fundamental functions of chaperonins are assisting proteins in acquiring and maintaining their activity under physiological conditions and initiating stress responses under stressful conditions. Chaperonins also indirectly regulate biological processes such as mitochondrial function, cytoskeleton organization, the cell cycle, immunity, autophagy, and apoptosis. Owing to the biological fundamentality and cross-species conservation of chaperonin functions, diseases associated with chaperonins—such as genetic disorders, neurodegenerative disorders, cardiovascular diseases, inflammatory diseases, autoimmune diseases, infectious diseases, and neoplastic diseases—can affect most eukaryotes throughout their entire lifespan and involve multiple systems and organs. Consequently, chaperonins have emerged as valuable non-invasive biomarkers for disease diagnosis and prognosis, as well as highly promising therapeutic targets for intervention. This article provides a detailed review of the current research status and progress regarding the pathogenic mechanisms of chaperonins in human diseases, related drug development, and clinical applications. It aims to offer basic researchers, drug developers, and clinicians a perspective on diseases through the lens of chaperonins, thereby promoting the translation of related research findings into clinical applications.

## Introduction

Exposure of cells and tissues to stressors can lead to cellular dysfunction, damage, and even cell death. In response to such stimuli, cellular systems activate stress response mechanisms to mitigate and repair the resulting damage. The exploration of cellular stress response mechanisms to heat stress can be traced back to an accidental discovery by Ritossa in 1962. He observed a novel pattern of chromosomal puffing in *Drosophila* salivary gland chromosomes following an unintended increase in incubator temperature. This unexpected finding revealed that cells activate a specific transcriptional program under heat stress, known as the heat shock response [1, 2]. In 1974, Tissieres et al. demonstrated that elevated temperature induces the synthesis of a distinct set of proteins in mammalian cells, now known as heat shock proteins (HSPs), marking the beginning of subsequent research on HSPs [3]. Regarding prokaryotic HSPs, Georgopoulos discovered in 1978 that the *groE* gene in *Escherichia coli* (*E. coli*) affects phage morphogenesis [4]. In 1980, Yamamori et al. found that heat stress could induce transcription of the *groE* gene in *E. coli*, confirming that groE is a HSP [5]. In 1981, Tilly et al. first identified that the *groE* gene locus in *E. coli* actually consists of two separate genes, *groEL* and *groES* [6]. In 1988, Hemmingsen et al. first proposed to categorize and name proteins evolutionarily homologous to GroEL as chaperonins [7]. In eukaryotes, Reading et al. first characterized the *HSP60* gene in yeast in 1989, demonstrating its high sequence homology with *groEL* from *E. coli* [8]. In 1990, Gupta discovered that mouse TCP-1 shares sequence homology with GroEL from *E. coli* and HSP60 from yeast [9]. In 1992, Frydman et al. purified and analyzed TCP-1 from bovine testes and first discovered that TCP-1 does not exist alone but, together with other structurally similar subunits, forms a high-molecular-weight ring complex named the TCP-1 ring complex (TRiC) [10]. These pioneering studies laid the foundation for understanding HSPs.

**Fig. 1:**
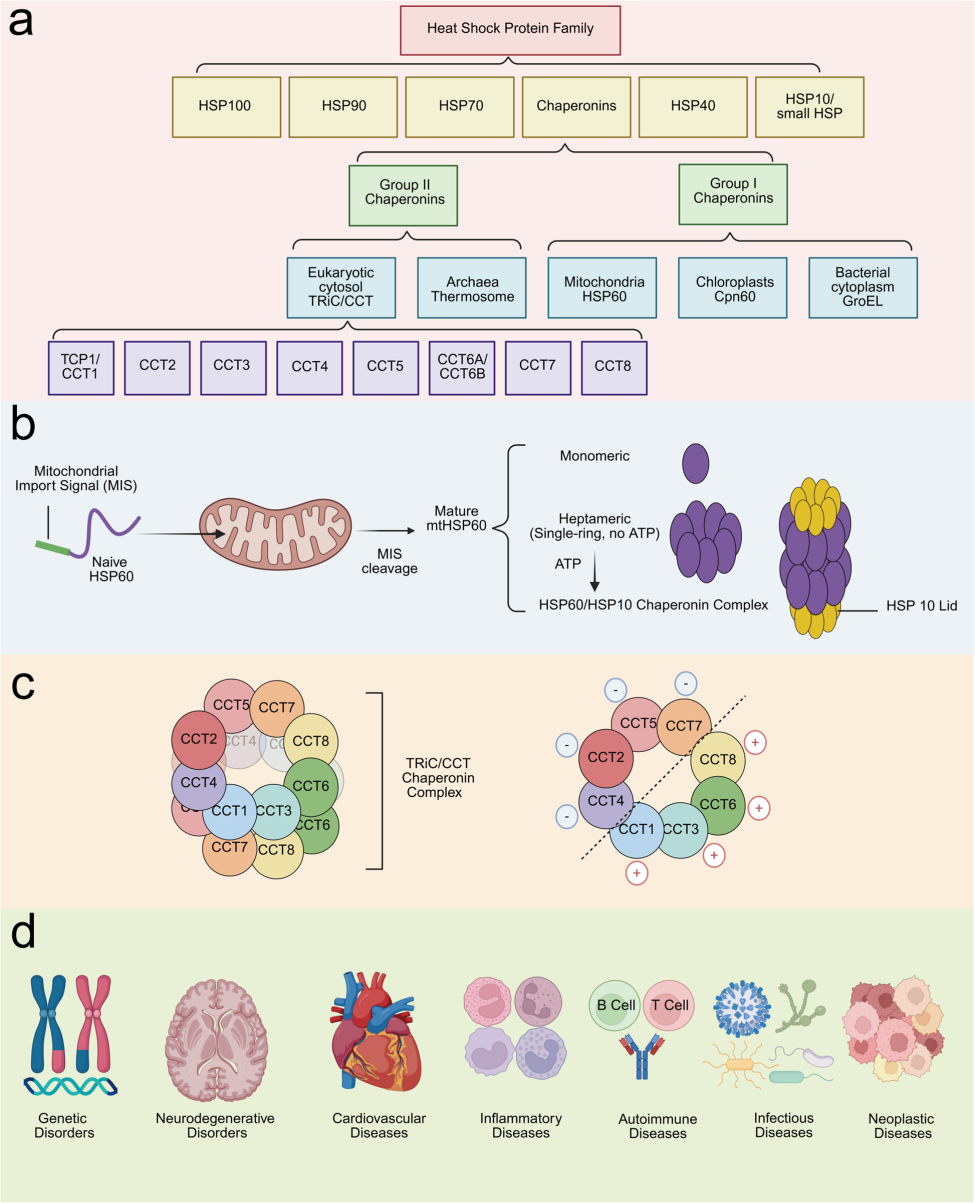
**a**​ Pedigree diagram of the heat shock protein family. In eukaryotic organisms, chaperonins mainly include HSP60 and TRiC, the latter comprising CCT1, CCT2, CCT3, CCT4, CCT5, CCT6, CCT7, and CCT8. **b**​ Structural diagrams of the chaperonins HSP60. Naive HSP60 carrying a mitochondrial import signal enters the mitochondria, where the MIS is removed to yield mature HSP60. This mature protein specifically exists in three forms: a monomeric form, a heptameric form, and a tetradecameric (14-mer) form. The tetradecameric HSP60 is a cylindrical structure composed of two stacked heptameric rings and requires the assistance of the co-chaperonin HSP10 for its protein-folding function. **c** Structural diagrams of the chaperonins TRiC/CCT. TRiC/CCT forms a cylindrical complex from two stacked octameric rings and functions independently without the need for a co-chaperonin. TRiC subunits can be divided into negatively charged hemispheres (CCT2, CCT4, CCT5, and CCT7) and positively charged hemispheres (CCT1, CCT3, CCT6, and CCT8). **d** Schematic diagram of disease spectrum of chaperonins. Chaperonins are primarily involved in the pathogenesis of genetic disorders, neurodegenerative disorders, cardiovascular diseases, inflammatory diseases, autoimmune diseases, infectious diseases, and neoplastic diseases

On the basis of the molecular weight of HSPs’ subunit/monomer, HSPs are classified into six major protein families: HSP100, HSP90, HSP70, Chaperonins, HSP40, and HSP10, as shown in Fig. 1a [11, 12]. Among these, chaperonins refer to HSPs with subunit/monomer molecular weights of approximately 55–65 kDa. Chaperonins are double-ring cylindrical complexes, with each ring comprising 7–9 subunits [13]. Each subunit can be divided into three domains: the equatorial domain, which contains the ATP-binding site and subunit-subunit contact points; the intermediate domain, which acts as a hinge region; and the apical domain, which is responsible for substrate recognition and binding [14, 15]. On the basis of their requirement for a co-chaperonin, chaperonins are classified into two groups. Group I chaperonins, such as mitochondrial HSP60, chloroplast Cpn60, and bacterial GroEL, form homo-heptameric rings and function with a co-chaperonin (e.g., HSP10 or GroES), as shown in Fig. 1b [16, 17]. Group II chaperonins, represented by the archaeal thermosome and eukaryotic cytosolic TRiC, typically assemble into hetero-octameric rings and facilitate substrate folding without the need for a co-chaperonin, as shown in Fig. 1c [15, 18]. Given the fundamental and conserved roles of chaperonins in protein folding and stress responses, diseases arising from mutations in chaperonin-encoding genes or dysregulated expression can manifest throughout the human lifespan and affect multiple physiological systems, as shown in Fig. 1d [11, 15, 19].

This review begins with an overview of the structural features, substrate proteins, folding mechanisms, and fundamental biological functions of eukaryotic chaperonins. Subsequently, following the chronology of embryonic development, it systematically elaborates on the biological roles of chaperonins starting from sperm and oocytes, through fertilization and embryogenesis, to the development of organs such as the brain, craniofacial structures, teeth, eyes, and heart. Next, it focuses on the mechanisms involving chaperonins in genetic disorders, neurodegenerative disorders, cardiovascular diseases, inflammatory diseases, autoimmune diseases, infectious diseases, and neoplastic diseases. Finally, it provides a detailed summary of the advances and challenges in chaperonin-related disease diagnosis and prognosis assessment, reviews the current therapeutic strategies and drug development, and offers an outlook on future directions for chaperonin-based therapies. Compared with previous reviews, this article covers a broader spectrum of chaperonin-related diseases and places greater emphasis on clinical applications, including diagnosis, treatment, drug resistance, and prognosis evaluation. It aims to provide a theoretical foundation for mechanistic exploration, clinical practice, and translational research of chaperonin-related diseases, and to offer clinical physicians, basic researchers, and drug developers a perspective on chaperonins. This is intended to encourage professionals across different fields to re-examine relevant topics and jointly promote the translation of research findings into clinical practice.

## Fundamental characteristics of eukaryotic chaperonins

This article primarily focuses on the role of chaperonins in human health and disease, and therefore we will concentrate on two eukaryotic chaperonins associated with human physiology and pathology, namely HSP60 and TRiC. Prior to providing a detailed review of the current research on their relevance to human health and disease, this chapter will first briefly introduce eukaryotic chaperonins basic characteristics, including structural features, substrate proteins, protein folding processes, and cellular functions.

### Structure of chaperonin

HSP60 is a mitochondrial chaperonin that also performs numerous other functions in the cytoplasm, cell membrane, extracellular environment, and body fluids [20]. The HSP60 (*HSPD1*) gene is located on human chromosome 2q33.1. In the cytoplasm, the newly translated, immature HSP60 protein (naive HSP60) contains an N-terminal mitochondrial import signal (MIS). Upon entry into the mitochondrion, MIS is cleaved, resulting in the formation of the mature HSP60 protein (mtHSP60) [20]. HSP60 can exist in monomeric, heptameric (single-ring), and tetradecameric (double-ring) forms [21]. The co-chaperone of HSP60, HSP10, typically exists as a heptamer composed of seven identical and symmetrical subunits [22]. In the absence of ATP, HSP60 primarily exists as a heptamer. Upon interaction with ATP and HSP10, HSP60 can polymerize to form a double-ring, tetradecameric cylindrical structure. HSP10 then acts as a lid, capping both ends of the cylinder and collectively forming a symmetrical HSP60/HSP10 complex, as shown in Fig. 1b [23].

TRiC is a hetero-oligomeric complex composed of two identical rings. Each ring of TRiC/CCT contains eight different subunits: CCT1 (TCP1), CCT2, CCT3, CCT4, CCT5, CCT6 (including isoforms CCT6A and CCT6B), CCT7, and CCT8, as shown in Fig. 1c [24, 25]. The TRiC subunits not only are integral components of hetero-oligomeric chaperonin but can also exist within the cell as free subunits or smaller oligomers [26]. The CCT1-8 subunits share approximately 30% sequence identity with each other, whereas each individual subunit​ shares approximately 60–70% identity across different species [27–32]. The assembly of TRiC is initiated by the CCT2, CCT4, CCT5, and CCT7 subunits, while CCT8, CCT1, CCT3, and CCT6 are individually involved in ring formation [33]. The subunits are arranged in a fixed order within the TRiC ring (CCT1-4–2–5–7–8–6-3) [24]. Based on their charge properties, TRiC subunits can be divided into negatively charged hemispheres (CCT2, CCT4, CCT5, and CCT7) and positively charged hemispheres (CCT1, CCT3, CCT6, and CCT8), which are involved in ATP hydrolysis and binding unfolded substrates, respectively [33]. Given that​ TRiC functions as an integrated complex, knockdown, deletion, or overexpression of any single subunit significantly impairs its overall function and affects the expression levels of the other subunits [34–36].

### Client substrates and folding process mediated by chaperonins

As a mitochondrial chaperonin, HSP60 is essential for the proper folding of proteins within the mitochondrion. Its primary substrates include metabolic enzymes, subunits of oxidative phosphorylation complexes, and proteins encoded by the mitochondrial genome [37]. HSP60 binds to unfolded proteins via hydrophobic interactions and, in an ATP-dependent manner, facilitates the folding of both nascent peptides and misfolded proteins [23]. The binding of ATP to the HSP60 heptameric ring triggers upward movement and torsion of the ring, resulting in significant expansion of the central cavity and providing a protected environment for substrate folding. The co-chaperonin HSP10 is recruited to both ends of the HSP60 tetradecamer via its apical domains after ATP binding, serving as a lid to encapsulate the cylindrical structure and initiate the folding process. Following ATP hydrolysis and the completion of protein folding, HSP10 dissociates, leading to opening of the cylinder and release of the folded substrate [20, 38, 39].

TRiC has broad binding specificity for hydrophobic structural features, particularly proteins with high β-sheet propensity and containing WD-repeats [14, 19, 40]. TRiC folds approximately 10% of the human proteome, with its most prominent substrates being actin and tubulin [14, 19, 34–36, 41–44]. The subunits of TRiC exhibit differential ATP affinities, with CCT1, CCT2, CCT4, and CCT5 displaying high affinity that enables ATP binding at physiological concentrations.​ These high-affinity subunits are all positioned on the same half of the TRiC ring, where they regulate an asymmetric power stroke to drive ATP hydrolysis [14]. ATP hydrolysis triggers the transition of TRiC from an open state to a closed state, forming a protective chamber conducive to protein folding. Upon completion of folding, the nucleotide-sensing loop of CCT3 detects the absence of γ-phosphate following ATP hydrolysis to ADP, which initiates a conformational change starting with the dynamics of the CCT3 apical protrusion and expands outwardly to the consecutive CCT6, CCT8, CCT7, and CCT5 subunits. Significant movements subsequently occur in CCT2, CCT4, and particularly the CCT1 subunits, leading to the opening of the TRiC ring and the release of the folded protein [33, 45–53].

### Cellular function of chaperonin

The fundamental functions of chaperonins in maintaining proteostasis primarily involve assisting proteins in acquiring and maintaining their activity under physiological conditions and initiating stress responses under stressful conditions. First, under physiological conditions, chaperonins essentially regulate biological processes, such as the cell cycle, cytoskeletal dynamics, cell growth and development, cell signaling, and mitochondrial homeostasis, by folding key proteins [54, 55]. Second, under stress conditions such as high temperature, viruses, ischemia, chaperonins are rapidly activated to protect cells from stress-induced damage. Chaperonins exert cytoprotective effect by participating in the refolding of denatured proteins, the proteasomal degradation of unstable or defective proteins, and the disassembly of oligomeric protein structures [54]. Beyond these two primary functions, chaperonins also possess activities that extend beyond traditional chaperone functions, including immunomodulation, inflammation regulation, autophagy regulation, oxidative stress modulation, apoptosis control, and intercellular communication, to maintain proteostasis. These non-canonical functions will be introduced in subsequent sections detailing the pathogenic mechanisms of chaperonin-related diseases [55–57].

## Chaperonins in embryogenesis

Embryogenesis refers to the entire developmental process from the fusion of sperm and oocyte to form a zygote, through the development of a complete embryo, until birth. During this earliest stage of life formation, chaperonins significantly influence the morphology and function of sperm, folliculogenesis, gamete fusion, embryo implantation, the sex of embryo, embryo survival, as well as organ development processes.

### Chaperonins in germ cell development

**Fig. 2:**
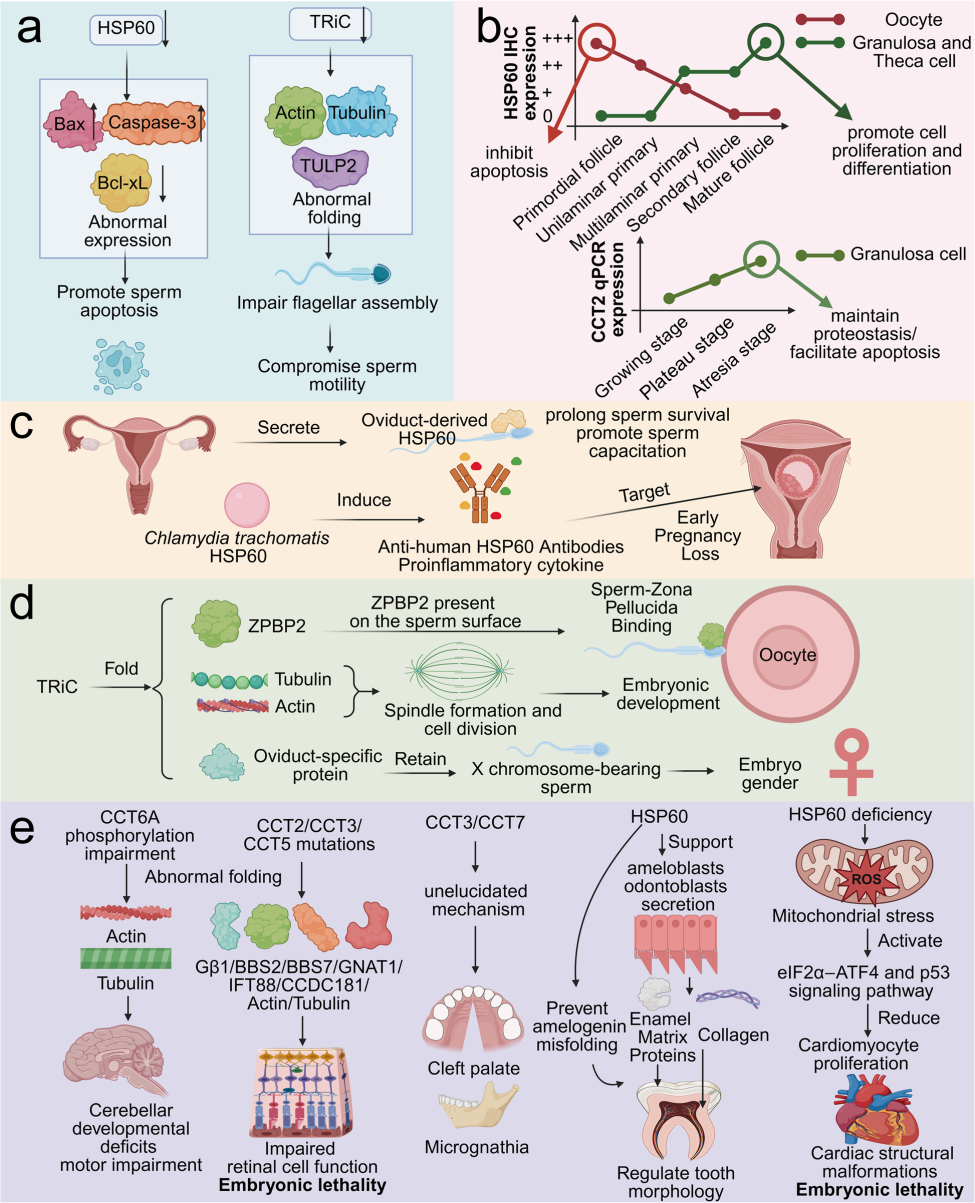
**a** Schematic diagram of chaperonins regulation in sperm development. Aberrant expression of chaperonins can lead to sperm apoptosis and abnormal flagellar development. **b** Schematic diagram of chaperonins regulation in folliculogenesis. Chaperonins participate in the proliferation, differentiation, and follicular atresia during follicular development. **c** Schematic diagram of HSP60 in embryonic development​. Endogenous HSP60 secreted by the fallopian tube can protect sperm, promote sperm survival, and facilitate fertilization. In contrast, HSP60 autoantibodies induced by exogenous HSP60 can impair embryo implantation, leading to early pregnancy loss. **d** Schematic diagram of TRiC in embryonic development​. TRiC mediates processes such as fertilization, embryonic development, and embryo sex selection by folding substrates including ZPBP2, tubulin, actin, and oviduct-specific proteins. **e** Schematic diagram of chaperonins in organ development​. Aberrant expression of chaperonins can lead to embryonic lethality or birth defects such as neurodevelopmental disorders, intellectual disability, ocular developmental abnormalities, craniofacial malformations, visual impairment, dental dysplasia, and cardiac development anomalies

HSP60 and TRiC are crucial regulators of sperm morphology, structure, quantity, and fertility. Consequently, mutations or aberrant expression of these chaperonins can lead to infertility by impairing spermatogenesis, as shown in Fig. 2a [58–60]. The downregulation of HSP60 expression promotes sperm apoptosis, by increasing the activity of the pro-apoptotic proteins Bax and Caspase-3 and decreasing the expression of the anti-apoptotic protein Bcl-xL. Conversely, the upregulation of HSP60 expression can increase sperm resistance to apoptosis [61–64]. A homozygous loss-of-function variant in *CCT6B* (c.615-2A > G) has been identified in patients with idiopathic non-obstructive azoospermia, in whom testicular tissue exhibits severe hypospermatogenesis associated with seminiferous tubule hyalinization. However, in the *Cct6b*⁻⁄⁻ mouse model, the normal fertility and intact testicular histology suggest that the *CCT6B* mutation may not directly cause non-obstructive azoospermia [65]. CCT8 regulates the intraflagellar transport system in sperm indirectly by folding TULP2.​ Knockdown of CCT8 leads to the formation of aberrant TULP2 aggregates in the cytoplasm, which disrupts intraflagellar transport, impairs flagellar assembly, compromises sperm motility, and can ultimately result in male infertility [66].

Chaperonins are key regulators of folliculogenesis, as shown in Fig. 2b. HSP60 expression in the oocytes of primordial follicle is highest during stages of follicular development—potentially providing cytoprotection against apoptosis—and gradually declines thereafter. In contrast, HSP60 expression in granulosa and theca cells increases with follicular development, where it correlates with cell proliferation and differentiation, while a decrease is associated with follicular atresia [67]. TRiC is also involved in the process of follicular development. Comparing the expression levels of CCT2 in bovine follicular granulosa cells from different stages, namely, the growing (G), plateau (P), and atresia (A) stages, revealed that CCT2 expression was significantly greater in granulosa cells from both the P and A stages than in those from the G stage. In the context of follicular atresia, where granulosa cells undergo large-scale apoptosis, the upregulation of CCT2 may represent a cellular stress response or pro-apoptotic response [68].

### Chaperonins in fertilization and early embryonic development

HSP60 plays a dual role with contrasting effects in both fertilization and embryo implantation, as shown in Fig. 2c. In cattle, baboons, and humans, near ovulation, HSP60 produced by oviduct epithelial cells is released into the lumen as a secretory protein and binds to sperm. HSP60 may contribute to a favorable microenvironment for sperm and eggs by prolong sperm survival, protecting sperm plasma membrane integrity, maintaining the functional integrity of sperm organelles and promoting sperm capacitation [69, 70]. However, pathogens such as *Chlamydia trachomatis* possess HSP60 with antigenic epitopes so similar to the human counterpart that​ infections in the female reproductive tract can sensitize the immune system against HSP60 protein. Consequently, the endogenous expression of HSP60 during embryonic development and decidualization can also trigger a pro-inflammatory milieu, characterized by​ the upregulation of IFN-γ and TNF-α and the secretion of HSP60-specific IgA/IgG antibodies, which directly interfere with implantation and result in early pregnancy loss [71].

TRiC and its subunits may affect the fertilization process, the initiation of embryonic development, and the sex of the embryo, as shown in Fig. 2d. Following capacitation, TRiC subunits (CCT2 and CCT6A) are significantly enriched in the periacrosomal region of the sperm head—the critical site for gamete interaction. By folding the client protein ZPBP2 (zona pellucida-binding protein 2) and facilitating its presentation on the sperm surface, TRiC indirectly mediates the specific binding of sperm to the zona pellucida, thereby promoting successful fertilization [72]. TRiC is one of the first proteins synthesized following zygotic genome activation. As early embryonic development requires extensive cytoskeletal remodeling—such as spindle formation and cell division—the expression of TRiC is likely to ensure the efficient folding of actin and tubulin under zygotic control, thereby supporting the initiation of embryonic development [13]. High expression of CCT4 in the oviducts of mother rabbits supplemented with calcium and magnesium was significantly positively correlated with an increased proportion of female offspring. CCT4 may promote the retention and storage of X chromosome-bearing sperm in the oviduct by assisting in the folding of oviduct-specific proteins and optimizing the survival environment for X sperm, thereby increasing the probability of female embryo formation [73].

### Chaperonins in organ development

Chaperonins are essential for the development of human organs, and their aberrant expression can lead to embryonic lethality or birth defects such as neurodevelopmental disorders, intellectual disability, ocular developmental abnormalities, craniofacial malformations, visual impairment, dental dysplasia, and cardiac development anomalies, as shown in Fig. 2e.

Regarding neural development, HSP60 is a fundamental chaperonin for brain development, showing sustained expression in the rat brain during postnatal development [74]. CCT6A, whose phosphorylation is regulated by ERK, participates in cerebellar neuronal development by folding the cytoskeletal proteins actin and tubulin. Consequently, impaired ERK-mediated phosphorylation of CCT6A leads to cytoskeletal folding defects, which disrupt granule cell migration, dendritic branching of Purkinje cell, and synapse formation, ultimately resulting in cerebellar developmental deficits and mild motor impairment [75]. Abnormal expression of CCT5 and CCT8 is also associated with intellectual developmental abnormalities in patients with Cri-du-chat syndrome and Down syndrome, respectively [48, 76, 77].

Regarding craniofacial development, Cct3 is expressed during early craniofacial development, with prominent localization in the mandibular portion of the first pharyngeal arch, supporting it as a candidate gene associated with orofacial clefting and micrognathia [78]. Furthermore, in a retinoic acid-induced cleft palate (CP) mouse model, CCT7 was identified as a potential key regulatory factor in the pathogenesis of CP and is a candidate gene contributing to the increased risk of CP [79].

Regarding ocular and retinal development, the aberrant expression or genes mutation of TRiC and its subunits significantly impacts eye and retinal development, affecting ocular morphology and vision. The zebrafish *cct2* L394H-7del mutation disrupt Cct2-mediated folding of Gβ1, a key regulator of cell cycle checkpoints. This disruption causes significant cell cycle abnormalities (extended S-phase duration and blocked M-phase transition), which in turn induce retinal cell apoptosis and lead to defective retinal development, and embryonic lethality at 5 days post-fertilization [80]. The mouse *Cct2* T400P mutant protein is structurally unstable, which leads to its increased affinity for the chaperone HSP90. This interaction is followed by rapid proteasomal degradation of the mutant protein, resulting in a loss-of-function of CCT2 and thereby contributing to the embryonic lethal phenotype [81]. The mouse *Cct2* R516H mutation impairs TRiC chaperonin function, causing a marked depletion of key substrate proteins (e.g., BBS2, BBS7, GNAT1) and the mislocalization of ciliary proteins (e.g., IFT88, CCDC181). This severe disruption of the photoreceptor connecting cilium’s transport and maintenance functions ultimately triggers photoreceptor apoptosis, resulting in the clinical phenotype of retinal degeneration and blindness [81]. Consistent with the combined effects of the two mutations described above,​ the compound heterozygous mice (T400P/R516H) died within two weeks after birth [81]. The zebrafish *cct3* gene deletion (143 bp deletion) may disrupts retinotectal development by impairing​ the folding of tubulin, thereby impeding​ retinal ganglion cells differentiation and damaging​ neurite extension and guidance [82]. The zebrafish U762 mutation (*cct5* ^U762/u762^) may hinder the compensatory growth capacity of the eye following *tcf7l1a* mutation, likely by impairing the folding of cytoskeletal proteins, thereby disrupting normal zebrafish eye growth and development [83]. Additionally, the zebrafish *cct5* tf212b (G422V) mutant presented decreased F-actin filaments in the retina, which could lead to retinal defects, as the numbers of retinal ganglion cells and amacrine cells are affected by decreased actin polymerization in the retina [84, 85].

Regarding tooth development, HSP60 is expressed early in odontogenesis, and its expression level changes dynamically throughout development. It supports the secretory functions of ameloblasts and odontoblasts, and prevents amelogenin misfolding, thereby playing a key role in regulating tooth morphology [86].

Regarding cardiac development, HSP60 is indispensable for embryonic heart formation. The absence of HSP60 in embryonic cardiomyocytes leads to disrupted mitochondrial proteostasis, subsequently inducing mitochondrial stress, which activates the eIF2α‒ATF4 pathway and p53 signaling, resulting in increased amino acid metabolism and cell cycle arrest. These cumulative effects ultimately cause reduced cardiomyocyte proliferation and abnormal cardiac morphology, and even embryonic lethality [87].

## Chaperonins in human diseases

Chaperonins continue to play important roles in the human body after embryogenesis and birth. Their gene mutations and aberrant expression are closely associated with a variety of human diseases, including genetic disorders, neurodegenerative disorders, cardiovascular diseases, inflammatory diseases, autoimmune diseases, infectious diseases, and neoplastic diseases, among others. This chapter will focus on elucidating the pathogenic mechanisms of chaperonins in these diseases, thereby laying the groundwork for subsequent discussions on their potential as disease biomarkers and therapeutic targets.

### Chaperonins and genetic disorders

**Fig. 3:**
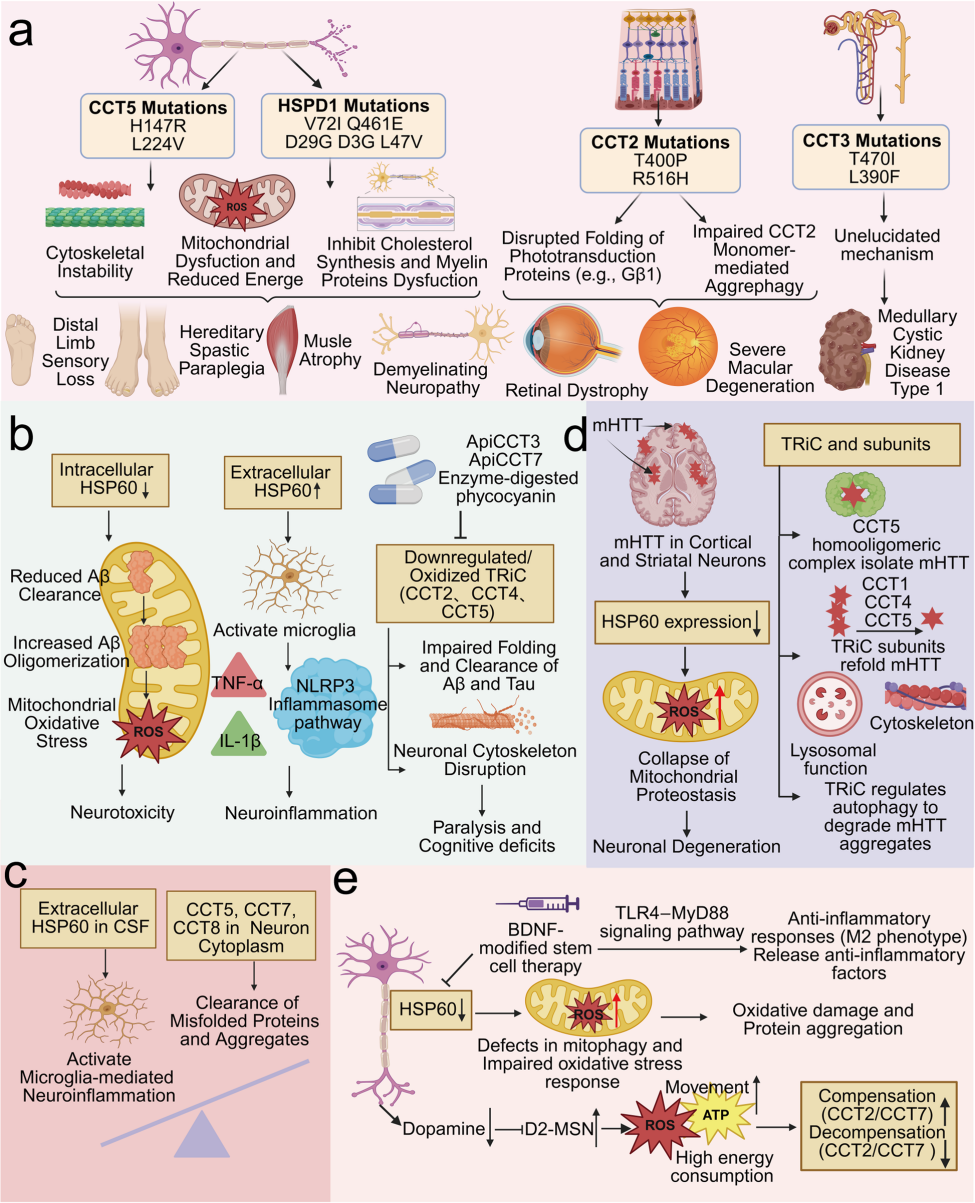
**a** Schematic diagram of chaperonins and genetic diseases. Mutations in chaperonin genes can lead to hereditary sensory neuropathy, hereditary motor neuropathy, hereditary demyelinating neuropathy, hereditary optic neuropathy, and hereditary nephropathy. **b** Schematic diagram of the pathogenic mechanisms of chaperonins in Alzheimer’s disease. Aberrant expression of chaperonins can lead to neurotoxicity and neuroinflammation, and is associated with manifestations such as paralysis and cognitive deficits. **c** Schematic diagram of the pathogenic mechanisms of chaperonins in amyotrophic lateral sclerosis. Dysregulation of HSP60 and TRiC can mediate microglial activation and remove misfolded proteins and aggregates, respectively, thereby influencing disease progression. **d** Schematic diagram of the pathogenic mechanisms of chaperonins in Huntington’s disease. Aberrant expression of chaperonins participates in the pathogenesis of Huntington’s disease by regulating mitochondrial homeostasis, and influencing the sequestration, folding, and autophagic degradation of mHTT. **e** Schematic diagram of the pathogenic mechanisms of chaperonins in Parkinson’s disease. Aberrant expression of HSP60 can lead to mitochondrial oxidative stress damage and protein aggregation, thereby contributing to the pathogenesis of Parkinson’s disease. The upregulation of CCT2 and CCT7 represents an adaptive response to oxidative stress-induced misfolding or damage of cytoskeletal proteins, whereas​ the tremorous motor phenotype of PD increases energy expenditure, ultimately inducing a decompensatory downregulation of CCT2 and CCT7 expression

Neurological genetic disorders represent a common category of genetic diseases associated with chaperonin mutations, encompassing sensory, motor, and demyelinating forms, as shown in Fig. 3a. Regarding sensory neuropathies, the *CCT5* H147R mutation causes mutilating hereditary sensory neuropathy with spastic paraplegia, characterized by distal limb sensory loss. The underlying pathogenic mechanism involves impaired TRiC assembly and folding, which subsequently disrupts neuronal cytoskeletal integrity and neural signaling processes [88–91]. Regarding motor neuropathies, *HSPD1* V72I and Q461E mutations are strongly linked to hereditary spastic paraplegia. These mutations primarily drive pathogenesis by disrupting HSP60 conformational dynamics, thereby impairing mitochondrial energy production and protein quality control, resulting in axonal degeneration that manifests clinically as lower limb spasticity and weakness [92, 93]. Additionally, the *CCT5* L224V mutation causes muscle atrophy, likely through the combined effects of cytoskeletal homeostasis dysregulation, denervation, inactivity, and malnutrition [94]. Regarding demyelinating neuropathies, three *HSPD1* mutations (D29G, D3G, and L47V) have been identified in leukodystrophy, which is a group of inherited diseases characterized by impaired formation or maintenance of cerebral myelin. The pathogenic mechanisms of these mutations primarily involve compromised chaperonin function, leading to misfolding of mitochondrial and myelin proteins coupled with inhibited cholesterol synthesis, thereby affecting the energy and raw materials essential for myelination/maintenance [95–98].

In ophthalmic genetic disorders, Leber congenital amaurosis (LCA) is an inherited, early-onset retinal dystrophy accompanied by severe macular degeneration. Compound heterozygous mutations *CCT2*-T400P and R516H identified in LCA patients disrupt both TRiC-mediated folding of key phototransduction proteins (e.g., Gβ1) and CCT2 monomer-mediated aggrephagy, ultimately leading to photoreceptor dysfunction and aberrant retinal development [52, 99, 100].

In renal genetic disorders, medullary cystic kidney disease type 1, an autosomal dominant tubulointerstitial nephropathy, is associated with two *CCT3* mutations: T470I and L390F [101].

### Chaperonins and neurodegenerative disorders

#### Alzheimer’s disease

Alzheimer’s disease (AD)​ is a progressive neurodegenerative disorder of the central nervous system characterized by cognitive dysfunction and behavioral impairments. The diagnosis of AD has shifted from traditional symptom-dependent criteria to biomarker-dependent criteria. Chaperonin dysfunction is directly linked to key neuropathological features of AD, including Aβ deposition, tau protein hyperphosphorylation, and loss of the mitochondrial membrane potential. HSP60 expression is elevated in the lymphocytes of patients with early-stage AD, and urinary CCT1 levels can help distinguish mild cognitive impairment (the prodromal stage of AD) patients from normal controls ​​ [102–104].

HSP60 has a dual pathogenic mechanism in AD pathogenesis. HSP60 is significantly upregulated in the lymphocytes and brain tissues of AD patients, likely representing a stress response to Aβ and tau pathology, which may exert a neuroprotective effect. However, once HSP60 function is impaired within neuronal mitochondria, it hinders the clearance of Aβ from mitochondria and the inhibition of Aβ oligomerization, exacerbating intramitochondrial oxidative stress and leading to Aβ-induced mitochondrial toxicity and neurotoxicity ​​ [57, 103]. On the other hand, extracellular overexpression of HSP60 can activate microglia (the key immune cells involved in neuroinflammation), modulate the expression of TNF-α and IL-1β, and engage the NLRP3 inflammasome pathway, thereby promoting neuroinflammatory responses, as shown in Fig. 3b [102, 105].

The pathogenic mechanism of TRiC in AD primarily involves impaired clearance of neuropathological aggregates due to its downregulation. For example, decreased expression of CCT2 and CCT4 in the AD brain may reduce the capacity to fold or clear tau and Aβ. CCT1 and CCT8 act as toxic inhibitors of Aβ, alleviating Aβ-induced paralysis phenotypes. The apical domains of CCT3 and CCT7 (ApiCCT3 and ApiCCT7) can also inhibit tau aggregation in a dose-dependent manner. In AD mouse models, the upregulation of CCT4 via the oral administration of enzyme-digested phycocyanin reduces Aβ aggregation and improves cognitive deficits [47, 106–108]. Beyond simple downregulation, oxidative modifications of TRiC also play a significant role in AD pathogenesis. Compared with that in control mice, the CCT5 protein in AD mice exhibited a greater degree of oxidation. Oxidation of CCT5 can disrupt chaperonin function, leading to the accumulation and aggregation of misfolded proteins. Furthermore, it can impair the folding of actin and tubulin, thereby damaging the neuronal cytoskeleton, as shown in Fig. 3b [109].

#### Amyotrophic lateral sclerosis

Amyotrophic lateral sclerosis (ALS) is a neurodegenerative disorder characterized by the progressive degeneration and death of motor neurons. Significant elevation of HSP60 levels has been observed in the cerebrospinal fluid (CSF) of ALS patients. Given that HSP60 is a key regulator of microglial activation, its upregulation in ALS-CSF may contribute to pathogenesis by promoting chronic microglial activation and subsequent microglia-mediated neuroinflammation [110]. In ALS models in which motor neuron-like cell lines express mutant *SOD1* genes, the CCT5, CCT7, and CCT8 subunits accumulate from the nucleus to the cytoplasm in neurons. This alteration in subcellular localization may represent an adaptive cellular response aimed at facilitating the clearance of misfolded proteins and aggregates generated during the disease process, as shown in Fig. 3c [111].

#### Huntington’s disease

Huntington’s disease (HD) is an autosomal dominant movement disorder that is clinically characterized by choreiform movements. The primary cause of HD is a mutation in the Huntingtin gene on chromosome 4, leading to the production of a mutant Huntingtin protein (mHTT) containing an expanded polyglutamine (PolyQ) repeat sequence. The aggregation of mHTT in cortical and striatal neurons results in progressive atrophy of the striatum and cerebral cortex, manifesting as motor dysfunction. mHTT can suppress HSP60 expression, which leads to the collapse of mitochondrial proteostasis and the accumulation of ROS, thereby accelerating neuronal degeneration, as shown in Fig. 3d [112].

TRiC and its subunits are involved in regulating mHTT aggregation through the following three mechanisms, as shown in Fig. 3d. First, the CCT5 homo-oligomeric complex can physically isolate mHTT by capping the ends of mHTT fibrils and encapsulating oligomers, thereby preventing further aggregation [113]. Second, CCT1, CCT4, and CCT5 can inhibit mHTT aggregation by facilitating the refolding of misfolded proteins [47, 114, 115]. Third, TRiC regulates autophagy by maintaining lysosomal function and the cytoskeleton, contributing to the degradation of mHTT aggregates [116]. Aberrant expression of TRiC and its subunits is associated with key neuropathological features of HD, including mHTT protein aggregation and corticostriatal atrophy. Therefore, maintaining normal TRiC expression has therapeutic value for ameliorating HD symptoms. Specifically, CCT3 and ApiCCT1 (the apical domain of CCT1) significantly reduce mHTT aggregate formation, improve both anterograde and retrograde transport of brain-derived neurotrophic factor, restore the trophic status of striatal and cortical neurons, and consequently exert neuroprotective effects [117–120]​​.

#### Parkinson’s disease

The primary pathological hallmarks of PD are progressive degeneration, apoptosis, and loss of dopaminergic neurons in the substantia nigra pars compacta. HSP60 is significantly lower in PD neurons, which coincides with defects in mitophagy and an impaired oxidative stress response, thereby rendering dopaminergic neurons more vulnerable to oxidative damage and protein aggregation [121, 122] However, in the context of BDNF-modified stem cell therapy, upregulated expression of HSP60 can modulate microglial function by activating the TLR4‒MyD88 signaling pathway, promoting anti-inflammatory responses (such as polarization to the M2 phenotype) and releasing anti-inflammatory factors (e.g., IL-10 and TGF-β), consequently alleviating neuroinflammation, as shown in Fig. 3e [122].

Dysregulated redox homeostasis, which involves oxidative and nitrative stress, is a significant contributor to dopaminergic neuronal loss in PD. Altered expression of TRiC and its subunits in PD often represents a manifestation of the cellular stress response. In an SH-SY5Y cell model of oxidative stress and neurodegeneration induced by the neurotoxin MPP +, the expression of CCT2 is upregulated [123]. Similarly, in an N27 cell model of H_2_O_2_-induced apoptotic cell death, CCT7 expression is increased [124]. As oxidative stress can lead to the misfolding or damage of cytoskeletal proteins, the upregulation of CCT2 and CCT7 is likely an adaptive response to assist in the correct folding or repair of these critical proteins, which is important for counteracting oxidative stress-triggered neuronal apoptosis. However, other studies have identified CCT2 and CCT7 as key proteins whose expression is downregulated in D2-type medium spiny neurons (D2-MSNs) of Parkinson’s disease patients [125]. In healthy individuals, dopamine inhibits D2-MSNs to facilitate movement. In PD, reduced dopamine secretion leads to decreased inhibition of D2-MSNs, resulting in overexcitation and the manifestation of the hypokinetic motor phenotype of PD. Since neuronal activity in D2-MSNs is highly energy-consuming and the synthesis and folding operations of TRiC are also energy-intensive processes, under conditions of severe energy depletion, cells must prioritize the allocation of limited energy resources to sustain the most fundamental vital activities to avoid immediate cell death. Therefore, the observed downregulation of CCT2 and CCT7 in D2-MSNs may represent a decompensation of the TRiC stress response in the context of excessive metabolic and proteotoxic pressure caused by the hyperexcited state of these neurons, as shown in Fig. 3e. Beyond oxidative stress, nitrative modifications of proteins by reactive nitrogen species are also implicated in PD. Since CCT1 has been identified as a target of protein nitration in the rat brain, aberrant nitrative modification of CCT1 may contribute to the progression of neurodegenerative diseases such as PD [126].

### Chaperonins and cardiovascular diseases

#### Atrial fibrillation

**Fig. 4:**
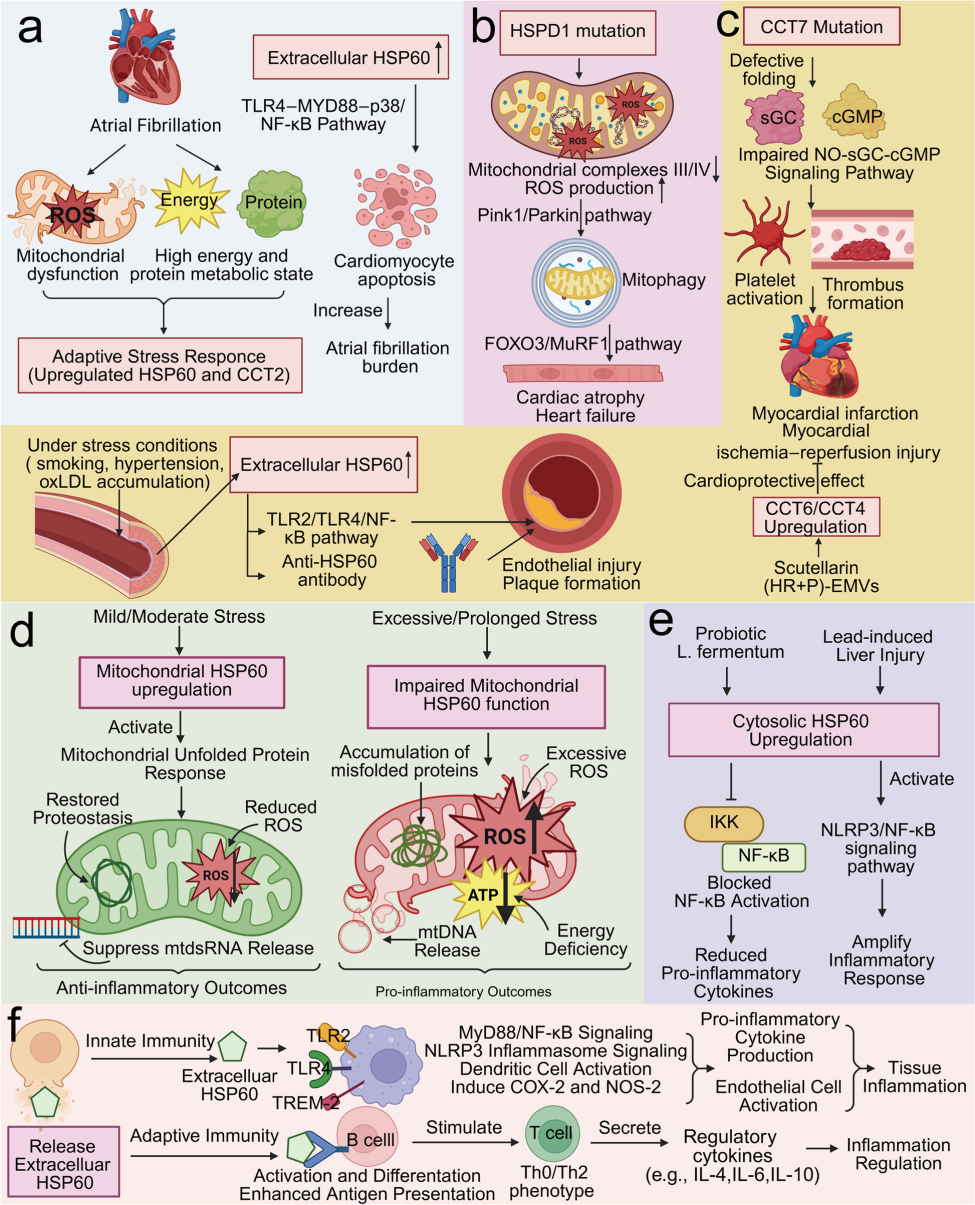
**a**​ Schematic diagram of chaperonins and atrial fibrillation. Atrial fibrillation can lead to mitochondrial dysfunction and a hypermetabolic state, which in turn induces a stress‑responsive upregulation of chaperonin expression. Extracellular HSP60 can trigger cardiomyocyte apoptosis, thereby increasing the cardiac load in atrial fibrillation. **b** Schematic diagram of chaperonins and dilated cardiomyopathy. Mutations in the *HSPD1* gene can disrupt mitochondrial homeostasis, triggering mitophagy and leading to cardiomyocyte atrophy, which ultimately results in heart failure. **c** Schematic diagram of chaperonins and coronary artery disease. Extracellular HSP60 can induce endothelial damage, contributing to the formation of atherosclerotic plaques. Mutations in the *CCT7* gene can disrupt the NO ‑ sGC ‑ cGMP signaling pathway, thereby promoting platelet activation and thrombosis formation, and subsequently leading to myocardial injury. **d** Schematic diagram of mitochondrial HSP60 in stress-intensity modulation of inflammation. When stress intensity exceeds the compensatory capacity of mitochondrial HSP60, it can disrupt mitochondrial homeostasis, leading to pro-inflammatory outcomes. **e** Schematic diagram of cytosolic HSP60 with context-dependent inflammatory outcomes. Under different contexts (*L. fermentum* or Lead exposure), both can induce the upregulation of cytosolic HSP60. However, their subsequent effects on inflammatory pathways differ, thereby leading to distinct inflammatory outcomes. **f** Schematic diagram of extracellular HSP60 in innate and adaptive immune orchestration. Extracellular HSP60 can induce innate immune responses through receptors such as TLR2, TLR4, and TREM-2, thereby promoting inflammatory progression and leading to tissue damage. Additionally, extracellular HSP60 can activate adaptive immune pathways, such as stimulating T cells via B cell activation, to secrete regulatory cytokines and modulate the progression of inflammation

Atrial biopsies from patients with atrial fibrillation (AF) reveal mitochondrial dysfunction, characterized by abnormal ATP levels, upregulation of mitochondrial stress chaperones, and fragmentation of the mitochondrial network [127]. Chaperonins can exert protective stress-response functions in AF. HSP60 expression is significantly elevated in the atrial tissue of patients with chronic AF [128]. Similarly, increased expression of CCT2 and other endolysosome-specific proteins was detected in atrial endolysosomes isolated from biopsy samples of goats with AF. The upregulation of chaperonins is likely related to the high energy and protein metabolic state during AF, potentially helping cardiomyocytes adapt to this heightened metabolic demand [129]. Furthermore, HSP60 is associated with reduced cardiac inflammation following radiofrequency ablation [130]. However, beyond this protective role, extracellular HSP60 can also induce cardiomyocyte apoptosis via the TLR4‒MYD88‒p38/NF-κB pathway and increase the AF burden, as shown in Fig. 4a [131].

#### Cardiomyopathy

Owing to the constant contractile work of the heart, its energy demands are enormous, making it highly susceptible to mitochondrial energy deficiency caused by HSP60 defects, potentially leading to cardiomyopathy and even heart failure. In a cardiomyocyte-specific *Hspd1* knockout mouse model, deletion of *Hspd1* altered the activity of mitochondrial complexes, the mitochondrial membrane potential, and ROS production, ultimately resulting in dilated cardiomyopathy (DCM), heart failure, and death [132]. Similarly, an *HSPD1* T320A missense mutation was identified in patients with familial DCM. This mutation causes a loss of HSP60 chaperone function, leading to reduced activity of mitochondrial complexes III/IV, increased ROS production, and activation of the Pink1/Parkin pathway, which enhances mitophagy and upregulates FOXO3/MuRF1 expression, consequently causing cardiomyocyte atrophy/fibrosis and impaired cardiac contractile function, as shown in Fig. 4b [133]. When patients with ischemic cardiomyopathy (ICM) or DCM were stratified into low and high-expression groups based on the median myocardial HSP60 expression level, the low HSP60 expression group required heart transplantation or LVAD implantation at a significantly younger age (average reduction of 4.9 years), suggesting that reduced HSP60 expression may accelerate the progression of heart failure [134].

#### Coronary artery disease

Chaperonins are associated with key pathological processes in coronary artery disease, including atherosclerotic plaque formation, coronary injury, and thrombus formation. HSP60 is directly involved in cardiovascular pathology, and elevated serum HSP60 levels are positively correlated with cardiovascular risk [135]. Serum HSP60 levels are significantly greater in chronic periodontitis patients who also have coronary heart disease than in those with periodontitis alone [136]. Furthermore, increased concentrations of anti-HSP60 antibodies are associated with greater coronary artery calcification in asymptomatic adults [137]. TRiC and its subunits also hold significant diagnostic value and contribute to the development of coronary artery disease. Kawasaki disease (KD), an acute systemic vasculitis syndrome primarily affecting children under five years of age, has coronary arteritis as its most severe complication. CCT1 is specifically expressed in coronary artery vascular smooth muscle cells, and its protein level is inversely correlated with the risk of coronary artery disease. Thus, CCT1 may serve as a marker for KD disease activity [138, 139]. Additionally, the heterozygous genetic mutation combination of *CCT7* Ser525Leu/*GUCY1A3* Leu163Phefs*24 significantly increases the risk of myocardial infarction. The *CCT7* Ser525Leu mutation leads to defective TRiC folding, severely impairing the protein levels and activity of soluble guanylyl cyclase (sGC) and cyclic guanosine monophosphate (cGMP). This results in dysfunction of the NO-sGC-cGMP signaling pathway, which normally exerts protective effects in the cardiovascular system, such as vasodilation and inhibition of platelet activation. The impaired NO-sGC-cGMP pathway accelerates thrombus formation, thereby significantly increasing the genetic susceptibility to myocardial infarction in carriers of these mutations [140, 141].

The upregulation of TRiC and its subunits can be beneficial in coronary artery disease. An endothelial cell hypoxia/reoxygenation (H/R) model was used to study myocardial ischemia‒reperfusion injury. Scutellarin has been shown to protect human cardiac microvascular endothelial cells from H/R injury by upregulating CCT6A, exerting a cardioprotective effect [142]. Additionally, Zhang et al. compared lncRNA expression profiles in endothelial microvesicles (EMVs) derived from human umbilical vein endothelial cells (HUVECs) after H/R injury (HR-EMVs) versus EMVs from HUVECs subjected to H/R with propofol post-conditioning (HR + P)-EMVs and reported that (HR + P)-EMVs highly expressed lncCCT4-2. HR-EMVs induce apoptosis and oxidative stress in cardiomyocytes, and the lncCCT4-2 from (HR + P)-EMVs, upon uptake by cardiomyocytes, bind to and stabilize CCT4 mRNA, slowing its degradation and enhancing CCT4 protein expression, significantly reducing oxidative stress and apoptosis in cardiomyocytes, improving cardiac function, and diminishing infarct size, as shown in Fig. 4c [143].

Coronary atherosclerotic heart disease, the most common form of coronary artery disease, is significantly driven by HSP60 during the atherogenic process. Under stress conditions such as smoking, hypertension, or oxLDL accumulation, HSP60 expression increases within arterial wall cells, and HSP60 is secreted extracellularly. Extracellular HSP60 acts as a damage-associated molecular pattern (DAMP), activating Toll-like receptors (TLR2/TLR4) and the NF-κB pathway, thereby promoting the secretion of pro-inflammatory cytokines such as IL-1β, TNF-α, and IL-6, exacerbating systemic inflammation and activating the vascular endothelium [144]. Concurrently, extracellular HSP60 can induce the production of anti-HSP60 antibodies, which mount an autoimmune attack against vascular endothelial cells, promoting endothelial injury and plaque formation, as shown in Fig. 4c [145, 146]. Furthermore, anti-HSP60 antibody levels are positively correlated with pulse wave velocity (an indicator of arterial stiffness) and negatively correlated with flow-mediated vasodilation (a measure of endothelial function) [147]. Therefore, reestablishing immune tolerance to HSP60 represents an important therapeutic direction for atherosclerotic plaque management.

### Chaperonins and inflammatory diseases

HSP60, a chaperonin with dual regulatory functions in both promoting and suppressing inflammation, exhibits activity that is dictated by its subcellular localization [57, 148]. In contrast, research on TRiC in inflammatory diseases remains relatively limited.

#### Mitochondrial HSP60 in stress-intensity modulation of inflammation

Within mitochondria, the pro- or anti-inflammatory effects of HSP60 are influenced by the intensity and duration of stress, as shown in Fig. 4d. The mitochondrial unfolded protein response (mtUPR) is a protective mechanism activated in response to mitochondrial stress. When misfolded or unfolded proteins accumulate within mitochondria, the mtUPR signaling pathway is initiated, upregulating the expression of mitochondrial chaperonins such as HSP60 to restore mitochondrial proteostasis and mitigate inflammation. In non-alcoholic fatty liver disease, HSP60 overexpression can suppress the release of mitochondrial double-stranded RNA (mtdsRNA), thereby alleviating inflammation mediated by the mt-dsRNA/TLR3/MDA5 pathway [149, 150]. In acute drug-induced liver injury caused by acetaminophen overdose, the mtUPR is activated, and mitochondrial HSP60 expression increases in a time-dependent manner, whereas serum ALT/AST levels decrease significantly over time. The upregulation of HSP60 coincides with the control of liver inflammation [151].

However, if excessive stress leads to mitochondrial HSP60 aberrant expression or impaired function, misfolded proteins cannot be cleared promptly, which exacerbates mitochondrial damage and initiates or exacerbates inflammation through mechanisms such as energy deficiency, excessive ROS production, and the release of mitochondrial DNA (mtDNA) [152, 153]. Under the pathological conditions of osteoarthritis, sustained or excessive activation of the mtUPR can exhaust the protective capacity of intra-mitochondrial HSP60, leading to a significant downregulation of HSP60 expression in knee joint tissue. When HSP60 function is insufficient, the collapse of mitochondrial proteostasis directly causes mitochondrial dysfunction and massive production of ROS, which activate inflammatory signaling pathways (e.g., NF-κB) and promote the release of inflammatory factors (e.g., IL-1β and TNF-α). These cytokines, in turn, further attack chondrocytes and mitochondria, creating a vicious pro-inflammatory cycle that accelerates cartilage degeneration ​​​​ [154, 155].

#### Cytosolic HSP60 with context-dependent inflammatory outcomes

Within the cell, the pro- or anti-inflammatory effects of HSP60 are influenced by the specific type of stress or pharmacological agent, as shown in Fig. 4e. In alcohol-related tissue injury, treatment with the probiotic *L. fermentum* upregulates cytosolic HSP60, which subsequently inhibits IKK and stabilizes IκB-α, thereby blocking the activation of the NF-κB signaling pathway and ultimately reducing the production of downstream pro-inflammatory factors such as TNF-α, IL-6, and MMP-9 [156]. Conversely, in lead-induced liver injury, cytosolic HSP60 released due to mitochondrial dysfunction can activate the HSP60/NLRP3/NF-κB signaling pathway, which promotes caspase-1 activation, leading to the expression of pro-inflammatory factors such as IL-1β, IL-18, and TNF-α, thereby amplifying the inflammatory response [157].

#### Extracellular HSP60 in innate and adaptive immune orchestration

Extracellularly, HSP60 can promote the progression of inflammation by initiating innate immune responses, as shown in Fig. 4f. Under abnormal stress conditions, HSP60 can translocate from the inside of the cell to the cytomembrane, or be released as a free molecule due to cell death, or be secreted from living cells via non-classical pathways (e.g., lysosomal vesicles) into the extracellular environment [158, 159]. Extracellular HSP60 is upregulated in various conditions, including smoking-related pneumonia [159, 160], chronic calculous cholecystitis [161], atopic dermatitis [162], splenic inflammation [163], adipose inflammation [164, 165], and neuroinflammation [57, 103]. As a DAMP, extracellular HSP60 can be recognized by pattern recognition receptors on immune cells (e.g., macrophages, monocytes, microglia, and dendritic cells), such as Toll-like receptors (TLR2 and TLR4) or TREM-2 (triggering receptor expressed on myeloid cells 2), which can activate downstream signaling pathways (e.g., MyD88/NF-κB). Furthermore, extracellular HSP60 promotes dendritic cell maturation and activation, enhances macrophage infiltration and polarization toward a pro-inflammatory (M1) phenotype, stimulates the production of pro-inflammatory cytokines (e.g., TNF-α, IL-1β, IL-6, IL-12, and IFN-γ), and contributes to NLRP3 inflammasome activation, thereby exacerbating inflammation [166, 167]. In blood vessels, increased HSP60 expression may also induce COX-2 and NOS-2, promoting endothelial cell activation, increasing vascular permeability and inflammatory cell infiltration, and aggravating tissue edema and fibrosis [161].

Extracellularly, HSP60 can further modulate inflammation by influencing the adaptive immune response, as shown in Fig. 4f. HSP60 directly induces the proliferation of naive B cells and upregulates the expression of MHC class II molecules and co-stimulatory molecules on the B-cell surface, thereby increasing their capacity as antigen-presenting cells. It promotes the differentiation of activated B cells toward an immunoregulatory phenotype characterized by the secretion of IL-10 and IL-6. HSP60 indirectly regulates T-cell responses through B cells. T cells stimulated by HSP60-activated B cells secrete increased levels of IFN-γ and IL-10, skewing the T-cell response toward the Th0 phenotype or exerting a balancing/regulatory effect [167]. In patients with rheumatoid arthritis, self-HSP60, upon uptake by antigen-presenting cells, is presented via MHC class II molecules to low-affinity autoreactive CD4 + T cells. These T cells tend to exhibit a Th2 phenotype, secrete anti-inflammatory cytokines such as IL-4 and IL-10, and inhibit inflammatory progression [168]. In cholecystitis, a positive correlation exists between the expression of HSP60 and IL-13 in the gallbladder wall, indicating that HSP60 may promote Th2 immune bias, thereby influencing the inflammatory process [161].

### Chaperonins and autoimmune diseases

Chaperonins exhibit aberrant expression in most autoimmune diseases. For example, HSP60 is upregulated in patients with multiple sclerosis [169], anti-HSP60 IgG3 antibodies are elevated in HLA-B27-positive spondyloarthritis patients [170], CCT1 is overexpressed in the muscle tissue of patients with sporadic inclusion body myositis [171], CCT1 and CCT2 are downregulated in ankylosing spondylitis [172, 173], and CCT6A is upregulated in the plasma of systemic lupus erythematosus (SLE) patients [174]. Concurrently, chaperonins induced autoantibodies are also dysregulated. Therefore, chaperonins and the antibodies they induce hold potential as biomarkers for disease diagnosis or monitoring.

#### Endogenous chaperonins induce autoimmune antibodies

**Fig. 5:**
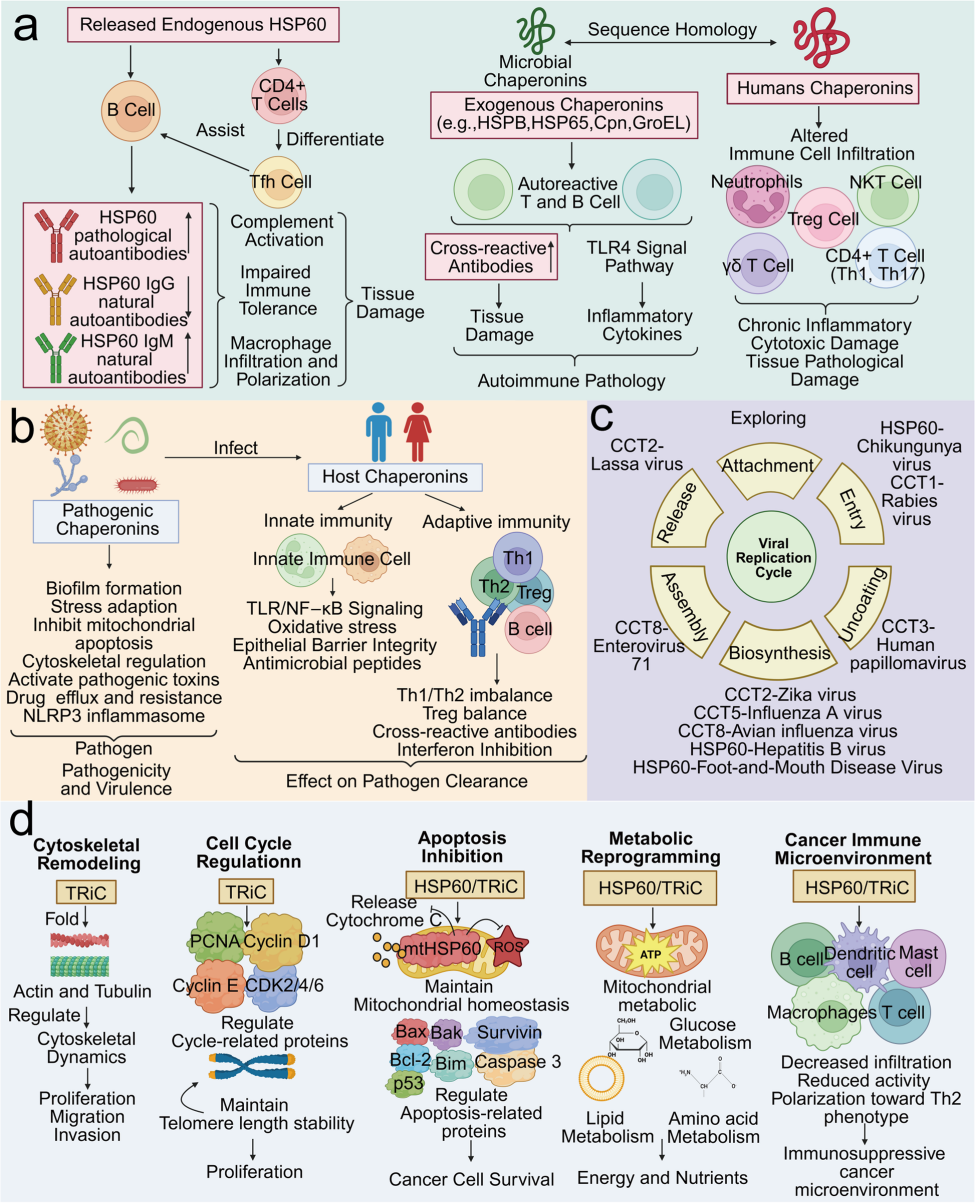
**a** Schematic diagram of chaperonins and autoimmune diseases. Endogenous HSP60 release can induce the production of autoantibodies against HSP60, leading to tissue damage. Exogenous pathogens carrying homologous chaperonin proteins may trigger the generation of cross-reactive antibodies, resulting in tissue injury. Chaperonins can also regulate the extent of immune cell infiltration, thereby participating in the progression of autoimmune diseases. **b** Schematic diagram illustrating the interaction between chaperonins, the host, and pathogenic microorganisms. Pathogenic microorganisms utilize chaperonins to maintain their pathogenicity and virulence during host invasion. The host employs chaperonins to mediate defense mechanisms; however, dysregulation of host chaperonin expression can lead to impaired pathogen clearance. **c** Diagram depicting chaperonin involvement in the viral replication cycle. Chaperonins participate in multiple stages of the viral replication cycle, including entry, uncoating, biosynthesis, assembly, and release. However, their involvement in the attachment step has not been reported to date. **d**​ Schematic diagram of the oncogenic mechanisms mediated by chaperonins. The primary oncogenic mechanisms of chaperonins involve cytoskeletal remodeling, cell cycle regulation, apoptosis suppression, metabolic reprogramming, and modulation of the tumor immune microenvironment

Endogenous chaperonins can be erroneously recognized by the immune system, leading to the production of pathological chaperonin antibodies, as shown in Fig. 5a. In RA, the synovial membrane produces and releases the autoantigen HSP60, which is recognized by B cells, leading to the production of anti-HSP60 autoantibodies that drive pathogenic autoimmunity, exacerbating inflammation and bone erosion [164, 175]. In atherosclerosis, extracellular HSP60 can induce the differentiation of HSP60-reactive CD4 + T cells into pathogenic T follicular helper cells, which assist B cells in producing anti-HSP60 antibodies. Anti-HSP60 antibodies can not only increase macrophage infiltration and polarization toward the M1 phenotype but also induce complement-dependent cytotoxicity or antibody-dependent cellular cytotoxicity, resulting in endothelial cell destruction and thereby promoting plaque formation [145, 146].

In addition to pathogenic autoantibodies, dysregulated expression of natural autoantibodies (nAAbs) also contributes to the pathogenesis of autoimmune diseases, as shown in Fig. 5a. In pregnant women with HT, nAAbs targeting HSP60 are produced. In healthy individuals, nAAbs recognize evolutionarily conserved antigens (e.g., heat shock proteins and mitochondrial enzymes) without causing pathological damage and are involved in maintaining immune tolerance. However, compared with healthy pregnant women, HT patients present elevated serum levels of anti-HSP60 IgM nAAbs and decreased levels of anti-HSP60 IgG nAAbs during both early and late pregnancy. Excess HSP60 IgM nAAbs may promote inflammation via complement activation, whereas a reduction in HSP60 IgG nAAbs could impair the maintenance of immune tolerance, adversely affecting the immune environment at the maternal‒fetal interface. Together, these imbalances may increase the risk of pregnancy complications such as miscarriage and preterm birth [176, 177].

#### Exogenous chaperonins induce cross-reactive antibodies

Exogenous chaperonin-induced cross-reactive chaperonin antibodies also contribute to the pathogenesis of autoimmune diseases, as shown in Fig. 5a. The heat shock protein HSPB from *Helicobacter pylori* (Hp) is highly immunogenic and shares homology with human HSP60. Consequently, the cross-reactive immune response triggered by Hp may lead to gastric mucosal damage [178]. In a mouse model of SLE, subcutaneous injection of bacterial HSP65 from *Mycobacterium leprae*—which shares high homology with self-HSP60—can accelerate SLE progression by cross-reactively activating autoreactive T and B cells and by activating TLR4 to promote the release of inflammatory cytokines [179]. The archaeon *Methanobrevibacter oralis* (*M. oralis*), a significant oral archaeon, expresses a Group II chaperonin, Cpn, which shares 28.8–40.0% sequence homology with human CCT subunits (CCT1–8). Antibodies generated against *M. oralis* Cpn can cross-react with human CCT proteins, potentially inducing autoimmunity and contributing to the pathogenesis of periodontitis [49, 50]. The chaperonin GroEL from the periodontal pathogen *Porphyromonas gingivalis* shares approximately 60% sequence homology with human HSP60 in immunodominant epitope regions. Therefore, anti-GroEL antibodies produced by the host immune system can attack human HSP60, particularly those that target cardiovascular tissues, leading to endothelial dysfunction and amplification of an inflammatory cascade, thereby participating in the formation and progression of atherosclerotic plaques [144]. Concurrently, periodontitis-induced tissue damage also releases HSP60 into the circulation and induces the production of anti-HSP60 antibodies, promoting plaque formation [145].

#### Chaperonins influence on autoimmune cell infiltration

Chaperonins contribute to the formation of a multi-layered autoimmune environment by influencing the infiltration of autoimmune cells, as shown in Fig. 5a. In patients with ankylosing spondylitis (AS), CCT2 expression is decreased in the blood. Given that CCT2 levels are negatively correlated with those of neutrophils and NKT cells, its low expression may promote the infiltration of these pro-inflammatory cells, thereby driving the chronic inflammatory process in AS [172]. Lupus nephritis (LN) is a severe complication of SLE. In LN patients, renal tubular epithelial cells express CCT6A, and γδ T cells can specifically bind and activate CCT6A, promoting the secretion of IFN-γ and IL-2, which causes cytotoxic damage to the renal tubules. Subsequent renal tissue injury releases soluble CCT6A, which in turn persistently activates more γδ T cells and creates a vicious cycle leading to more severe pathological damage in the kidney, thereby worsening LN [174]. In a rat model of RA, a wild-type peptide of the human HSP60 protein induced pro-inflammatory CD4 + FoxP3- T cells (such as Th1 and Th17 cells) but failed to induce regulatory T cells (Tregs), which led to a Th17/Treg imbalance, promoting the secretion of pro-inflammatory cytokines such as IFN-γ and IL-17 and exacerbating inflammation [180]. In type 1 diabetes, HSP60 acts as an autoantigen to activate pathogenic Th1 cells and concurrently engages the innate immune system via TLR-4 pathways. Together, these responses drive islet inflammation, culminating in β-cell destruction and insulin secretory failure [181].

### Chaperonins and infectious diseases

During the invasion of the host by exogenous pathogens, chaperonins can be hijacked by the pathogens to participate in their pathogenic processes while also responding to stress signals and collaborating with the immune system to increase the host’s anti-pathogen capacity, as shown in Fig. 5b. As chaperonins have been more extensively studied in human and zoonotic viral infections, we categorize pathogen-induced infectious diseases into viral diseases and non-viral diseases for discussion.

#### Human viruses and zoonotic viruses

##### Chaperonins and viral replication cycle

During viral infection, chaperonin expression is significantly upregulated.​ For example, HSP60 is upregulated during dengue virus infection, blood HSP60 levels are elevated in HIV patients, CCT5 is increased during influenza A virus infection, CCT8 is upregulated upon H9N2 avian influenza virus infection, and serum CCT1 levels rise during hepatitis C virus infection. These changes can serve as biomarkers to aid in the diagnosis and monitoring of viral infections [182–185]. Beyond their diagnostic importance, the upregulation of host chaperonins is a key step in the mechanism by which viruses hijack the host cellular machinery to promote infection. The roles of chaperonins in viral infection are shown in Fig. 5c.

In the entry stage, HSP60 is an interacting protein of the Chikungunya virus and may function as a viral receptor or co-factor, participating in the initial steps of viral entry into host cells [186]. In cells infected with the rabies virus (RABV), the host CCT1 relocalizes to Negri bodies (sites of RABV replication and transcription) and facilitates the co-transfection of viral proteins such as the nucleoprotein and phosphoprotein into cells, thereby promoting viral replication and transcription [187].

In the uncoating stage, the capsid of the human papillomavirus (HPV) consists of two structural proteins, L1 and L2. The host CCT3 directly interacts with the N-terminus of the HPV-16 L2 capsid protein, promoting capsid dissociation to release the viral genome, thus facilitating HPV-16 infection [188].

The biosynthesis stage is a major phase where chaperonins participate in viral infection. In Foot-and-Mouth Disease Virus (FMDV) infection, HSP60 inhibits the degradation of viral non-structural proteins 3A and 2C by blocking caspase-mediated apoptosis and via the autophagy‒lysosome pathway, respectively, thereby stabilizing their protein levels. Stable viral non-structural proteins 3A and 2C promote the formation of the viral replication complex, enabling efficient RNA replication and assembly of FMDV [189]. During Hepatitis B virus (HBV) infection, HSP60 activates hepatitis B virus polymerase, promoting viral genome replication [190]. In Mosquito-borne Zika virus (ZIKV) infection, the host CCT2 protein interacts with the viral non-structural protein NS1, participating in viral replication [191]. Influenza A virus (IAV) infection significantly increases the expression level of CCT5 in host cells. Increased expression of CCT5 can promote the nuclear export of the viral nucleoprotein and increase viral RNA polymerase activity, thereby facilitating IAV replication [182]. The H9N2 avian influenza virus induces the upregulation of host CCT8, and overexpressed CCT8 interacts with the polymerase basic protein 2 to increase viral replication [183].

In the assembly stage, host CCT8 is recruited by RAB11A to viral replication organelles during enterovirus 71 replication, and CCT8 facilitates the conformational maturation of the pro-virion protein VP0 by folding it, increasing its susceptibility to cleavage by viral proteases into the mature viral proteins VP2 and VP4 [192].

In the release stage, the LASV matrix protein LASV-Z interacts with host CCT2, interfering with TRiC function, which disrupts the folding of actin and tubulin, causing cytoskeletal abnormalities. The disrupted cytoskeleton impedes the transport of lysosomal enzymes from the Golgi apparatus to lysosomes and inhibits the fusion of autophagosomes with lysosomes, leading to the accumulation of autophagosomes. LASV can exploit these non-degraded autophagosomes to promote the egress of virus-like particles [193].

##### Chaperonins and viral immune evasion

Chaperonins assist viruses in achieving immune evasion by modulating host Tregs, memory T-cell and interferon. During HBV infection, the serum level of soluble HSP60 (sHSP60) in patients with chronic hepatitis B is positively correlated with the HBV DNA level. sHSP60 can enhance the function of HBcAg-specific Tregs, leading to immunosuppression and facilitating persistent viral infection [194]. In HIV-1-infected individuals, HSP60 in CD4 + memory T cells regulates glutaminolysis to generate ATP to support IL-21 production, and in CD8 + memory T cells, it regulates fatty acid oxidation to generate ATP to support cytotoxic CD8 + memory T-cell responses, thereby assisting in the clearance of virus-infected cells. Impaired HSP60 expression may lead to dysfunctional virus-specific T-cell responses [195]. In Dengue virus infection, increased HSP60 expression can suppress the production of the antiviral IFN-α, indirectly enhancing viral replication [196]. H5N1 viral ribonucleoprotein competes with CCT4 to bind to CRM1 (a nuclear export receptor), interfering with the efficient nucleocytoplasmic transport of IFN-α mRNA, reducing IFN-α secretion, facilitating viral immune evasion, and leading to peribronchial and alveolar inflammatory cell infiltration, resulting in viral pneumonia [197]. The minor capsid protein pVI of human adenovirus type 5 (HAdV-C5) targets the mitochondrial membrane, causing the release of mtDNA and HSP60 into the cytoplasm. Cytosolic HSP60 then binds to cyclic GMP-AMP synthase, inhibiting the TBK1-IRF3 phosphorylation cascade and subsequent IFN-β gene transcription, thereby helping HAdV-C5 evade host immunity and promoting viral survival and replication [198].

##### Chaperonins and host pathological damage

During viral infection, chaperonins contribute to host pathological damage by mediating apoptosis and inflammasome pathways. In Coxsackievirus B3 (CVB3) infection, exosomal HSP60 not only promotes viral spread but also induces cardiomyocyte apoptosis, exacerbating CVB3-induced pathological damage [199]. In Japanese encephalitis virus (JEV) infection, HSP60 mediates microglial IL-1β production by regulating the NLRP3 inflammasome pathway, exacerbating neuroinflammation [200].

#### Non-viral pathogenic microorganisms

Beyond their roles in viral diseases and host antiviral immunity, chaperonins also play critical roles in the pathogenesis of infections caused by bacteria (*Helicobacter pylori*, *Clostridium difficile*, *Bordetella pertussis*, *Salmonella enterica*, *Shigella flexneri*, *Listeria monocytogenes*), chlamydia (*Chlamydia trachomatis*), fungi (*Histoplasma* spp., *Paracoccidioides* spp., *Candida albicans*, *Trichophyton indotineae*), and parasites (*Plasmodium falciparum*, nematode *Heligmosomoides polygyrus*).

##### Chaperonins and pathogen pathogenicity​ and virulence

Chaperonins contribute to pathogen survival and adaptation to stressful environments. In fungi such as *Histoplasma* spp. and *Paracoccidioides* spp., HSP60 mediates macrophage phagocytosis by binding to complement receptor 3; however, it suppresses phagocyte activation, leading to a low-inflammatory response and immune evasion, thereby promoting biofilm formation. Simultaneously, HSP60 helps maintain mitochondrial proteostasis and assists fungi in adapting to host-induced stress [201]. During Hp infection, extracellular vesicles released from infected cells exhibit significantly increased HSP60 expression. This elevated HSP60 can regulate the Bcl2/Bax expression balance, inhibiting the mitochondrial apoptosis pathway and promoting the survival of Hp-infected cells [178]. HSP60 and CCT7 are SUMOylation substrates in *Candida albicans*, and SUMOylation of chaperonins is potentially involved in cytoskeletal regulation and stress adaptation of the fungus [202]. While a *Candida albicans* strain with a single *cct8* allele deletion shows no growth defect, complete knockout of both *cct8* alleles leads to fungal death, indicating that CCT8 is essential for pathogen survival and represents a potential antifungal target [203].

Chaperonins are involved in activating the enzymatic activity of pathogenic toxins. The CCT4/CCT5 subunits directly interact with the glycosyltransferase domains of the toxins TcdA and TcdB from *Clostridium difficile*, promoting their folding and increasing their enzymatic activity [204]. During *Bordetella pertussis* infection, host CCT5 assists in refolding the PTS1 subunit (the enzymatic subunit of *pertussis* toxin), restoring its enzymatic activity, which leads to ADP-ribosylation of Gαi, disruption of intracellular cAMP signaling, and subsequent airway inflammation [205].

Chaperonins are involved in enhancing pathogen drug resistance. In refractory skin infections caused by *Trichophyton indotineae*, high expression of HSP60 may act synergistically with efflux transporters (e.g., MDR3) to promote the efflux of azole and allylamine antifungal drugs, thereby increasing drug resistance. Consequently, inhibiting HSP60 function could sensitize resistant strains and improve the efficacy of existing antifungals [206].

##### Chaperonins and host immune regulation

Chaperonins influence the host’s innate immunity.​ During *Listeria monocytogenes* infection, HSP60 acts as a receptor for the *Listeria* adhesion protein (LAP). The LAP‒HSP60 interaction activates the NF‒κB‒MLCK axis, triggering the endocytosis of the intercellular junctional complex in enterocytes, which increases paracellular permeability and ultimately facilitates the paracellular translocation of *Listeria* [207, 208]. Infection with *Salmonella enterica* enteritidis can significantly suppress HSP60 protein expression in intestinal tissue. The decreased expression of HSP60 is associated with increased oxidative stress and the upregulation of pro-inflammatory factors, destroying the host intestinal mucosal barrier [209]. Following infection of *Caenorhabditis elegans* with *Shigella flexneri*, Cct2 expression is significantly upregulated, promoting the nuclear localization of DAF-16, which activates the DAF-2/DAF-16 insulin signaling pathway and induces the expression of antimicrobial peptides [210]. In the whole-blood transcriptome of children with severe malarial anemia caused by *Plasmodium falciparum* infection, the HSP60, HSP70, and TLR2/4 signaling pathways were significantly downregulated. This leads to reduced expression of MyD88, IRAK4, and NF-κB and decreased production of inflammatory cytokines (e.g., IL-6 and IL-1β), resulting in aberrant innate immune activation, which impairs the host’s ability to effectively clear the malaria parasite and exacerbates erythropoietic dysfunction and hemolysis [211]. In pigs infected with *Salmonella enterica*, those with the G/A genotype for the *CCT7* SNP (AK240296.c1153G > A) presented increased serum IFN-γ levels compared with those in G/G homozygotes. The G/A genotype promotes non-specific infiltration of inflammatory cells such as neutrophils and monocytes, aggravates tissue damage, facilitates bacterial dissemination, and increases fecal bacterial shedding [212].

Chaperonins influence the host’s adaptive immunity.​ When *Chlamydia trachomatis* (Ct) infects female reproductive tract cervical epithelial cells, Ct-HSP60 is released into host tissues. Owing to its high immunogenicity, Ct-HSP60 triggers a Th1-type response, promoting the secretion of pro-inflammatory cytokines such as IFN-γ, which exacerbates acute tissue inflammatory damage. Persistent exposure to Ct-HSP60 can also induce immune tolerance, leading to a Th1/Th2 imbalance skewed toward an anti-inflammatory response (e.g., elevated IL-10), which suppresses pathogen clearance and results in chronic inflammation, fibrosis, and scarring of the fallopian tube epithelium. Furthermore, because Ct-HSP60 shares homologous sequences with human HSP60, it may induce cross-reactive antibodies that attack the host’s fallopian tubes, worsening tissue damage and contributing to tubal factor infertility and pelvic inflammatory disease [213]. During infection with the nematode *Heligmosomoides polygyrus*, CCT8 contributes to effective anti-parasitic immunity by recruiting effector cells (e.g., alternatively activated macrophages, eosinophils), participating in T-cell maturation, selection, and activation, regulating Th2 polarization, promoting antibody production, and maintaining the Treg balance [214].

### Chaperonins and neoplastic diseases

#### Chaperonins and cancer phenotypes

HSP60 and TRiC exhibit upregulated expression and enhanced chaperonin activity in the majority of cancers and/or their extracellular vesicles. This has been reported in a wide range of cancers, including glioma [215], neuroblastoma [216], medulloblastoma [217], sinonasal adenocarcinoma [218, 219], nasopharyngeal carcinoma [219], oral squamous cell carcinoma [220–222], ameloblastoma [223], head and neck squamous cell carcinoma [224, 225], papillary thyroid carcinoma [226–229], laryngeal cancer [230], esophageal squamous cell carcinoma (ESCC) [231–239], lung cancer [240–253], breast cancer [25, 35, 254–272], gastric cancer [19, 223, 273–277], hepatocellular carcinoma (HCC) [34, 36, 223, 278–292], gallbladder cancer [293], pancreatic cancer [153, 294, 295], renal cell carcinoma [296], colon cancer [223, 297–308], endometrial cancer [309], cervical cancer [310–314], ovarian cancer [315–321], bladder cancer [322, 323], prostate cancer [223, 324–329], testicular cancer [330, 331], acute myelocytic leukemia [332], B-cell non-Hodgkin’s lymphoma [333–335], multiple myeloma [336–338], leiomyosarcoma [339], melanoma [340], osteosarcoma [341–343], and Ewing’s sarcoma [344]. In summary, the expression levels of these chaperonins are closely correlated with malignant biological phenotypes of cancer cells—such as proliferation, migration, and invasion—as well as malignant clinical phenotypes, including advanced tumor stage, higher pathological grade, metastasis, recurrence, shortened survival, and treatment resistance.

However, exceptions exist. For example, HSP60 expression is downregulated in ovarian cancer cells derived from ascites [345], in some cases of advanced colorectal cancer [346], and in certain HCCs [347]. Similarly, CCT3 expression is decreased in patients with testicular cancer exhibiting asthenospermia [348], and CCT4 expression is downregulated in Wilms’ tumor tissue [349]. In these specific contexts, the downregulation of chaperonin expression is associated with more malignant tumor phenotypes.

#### Chaperonins and carcinogenic mechanisms

Aberrantly expressed chaperonins contribute to tumorigenesis and progression by participating in various biological processes, including cytoskeletal remodeling, cell cycle regulation, apoptosis, metabolic reprogramming, and modulation of the tumor immune microenvironment, as shown in Fig. 5d.

Regarding cytoskeleton, chaperonins, particularly TRiC, promote the occurrence and development of cancer by regulating cytoskeletal dynamics. For example, CCT2 in neuroblastoma [216] and breast cancer [350], CCT4 in pre-B cell acute lymphoblastic leukemia [351] and ESCC [233, 234, 236], and CCT8 in glioma [215] can facilitate the folding of cytoskeletal proteins (actin and tubulin), thereby stabilizing the cytoskeleton, which drives malignant phenotypes that depend on the cytoskeleton, such as the proliferation, migration, and invasion of cancer cells.

Regarding cell cycle, chaperonins promote cancer proliferation by regulating the cycle-related proteins [352]. For example, CCT2 and CCT3 in breast cancer [266, 267, 350], CCT3 in gastric cancer [19] and melanoma [340], CCT8 in non-Hodgkin’s lymphoma [333], and CCT8 in HCC [288, 353–356] can all modulate the expression and activity of cell cycle-related proteins such as PCNA, Cyclin D1, Cyclin E, CDK2, CDK4, and CDK6, which drive cell cycle progression and enhance cancer cell proliferation. Additionally, chaperonins contribute to cancer proliferation by regulating the expression of telomere-associated proteins and modulating telomerase activity. For example, CCT4 in acute myeloid leukemia (AML) ​​ [357] and CCT6A in cervical cancer [312, 313] can maintain telomere length stability, thereby increasing the proliferative capacity of cancer cells and promoting cancer survival and progression.

Regarding apoptosis, chaperonins inhibit cancer apoptosis and promote carcinogenesis and progression by regulating apoptosis-related proteins such as Bax, Bak, Bcl-2, Bim, Caspase-3, Survivin and p53. As a mitochondrial chaperonin, HSP60 participates in maintaining mitochondrial homeostasis. HSP60 deficiency leads to dysfunctional mitochondrial metabolic enzymes, mtDNA leakage, decreased mitochondrial membrane potential, increased ROS production, and ultimately induces cancer apoptosis [358]. In the mitochondria of cancers, HSP60 is overexpressed and can directly bind to cyclophilin D to antagonize cyclophilin D-induced opening of the mitochondrial permeability transition pore, preventing cytochrome c release and caspase activation and thereby inhibiting cancer apoptosis [358]. In ameloblastomas, HSP60 overexpression helps cancer cells maintain mitochondrial homeostasis, limits excessive ROS production, and enables cancer cells to evade ROS-triggered apoptotic signals, thereby promoting cancer progression and recurrence [223]. In oral squamous cell carcinoma, HSP60 interacts with Survivin to form a complex, stabilizing mitochondrial Survivin and subsequently inhibiting caspase activation, thus suppressing cancer apoptosis [222]. Chaperonins also participate in regulating apoptosis by interfering with the cancer suppressive function of p53. In colon cancer cells, CCT8 binds to wild-type p53 in the cytoplasm, preventing its nuclear translocation [304]. CCT8 can also directly bind to the ribosomal protein RPL4 in the cytoplasm of colon cancer cells, disrupting the RPL4-MDM2 interaction and enhancing MDM2-mediated ubiquitination and degradation of p53 [303]. In cervical cancer, CCT8 forms a stable complex with the HPV E6 oncoprotein, increasing its ability to degrade p53 [314]. The obstruction of nuclear translocation or degradation of p53 can drive the expression of downstream cyclins, accelerate genomic instability, and promote tumor cell escape from apoptosis, thereby facilitating tumor proliferation, invasion, and metastasis.

Regarding cancer metabolic reprogramming, most malignant cancers require nutrients and energy, such as glucose, lipids, amino acids, and nucleotides, to support cell growth and proliferation. To survive under stressful conditions such as hypoxia and nutrient limitation, cancer cells initiate metabolic reprogramming. Mitochondrial metabolic reprogramming is a core component of cancer metabolic reprogramming. HSP60, a key molecule in maintaining mitochondrial metabolic function, promotes ATP synthesis through its involvement in folding mitochondrial metabolic enzymes and maintaining mitochondrial homeostasis, thereby supplying energy for cancer cells [253, 359, 360]. Chaperonins also regulate the metabolic reprogramming of glucose, amino acid, and lipid metabolism, thereby promoting cancer progression. In glucose metabolism, for example, the CCT6A/CCT8/THOC3/PFKFB4 axis in lung squamous cell carcinoma [361], the CCT6A/STAT1/HK2 axis in lung adenocarcinoma [248], CCT3 in lung adenocarcinoma [243], and CCT8 in cervical cancer [314] all regulate glycolytic enzymes, promoting cancer cell glycolysis and providing more energy for cancer cell proliferation. In amino acid metabolism, the activated CCT6A/RPS3/serine axis can provide material and energy for the rapid proliferation of HCC cells by mediating one-carbon metabolism [362]. Lipid metabolism, for example, the CCT3-LINC00326 axis in HCC [363] and CCT8 in cervical cancer [314], can influence oxidative stress levels and the energy supply within cancer cells by regulating lipid metabolism, thereby promoting cancer progression.

Regarding cancer immune microenvironment, the expression of chaperonin is associated with the extent of infiltration and polarization of anti-tumor immune cells. In lung adenocarcinoma, patients with high HSP60 expression levels show lower levels of infiltration by activated and immature B cells and CD4 + T cells within cancers [364]. In colon cancer, HSP60 can activate the TLR2‒MYD88 pathway, induce the differentiation of monocytic myeloid-derived suppressor cells, and inhibit T-cell activity [365]. In medulloblastoma, CCT2 expression is negatively correlated with the level of CD4 + T-cell infiltration [217]. In breast cancer, CCT2 can suppress CD4 + T-cell activation by restricting Ca2 + -NFAT1 signaling [366]. In lung adenocarcinoma, CCT3 can promote Th2 cell infiltration while inhibiting the activity of immature dendritic cells and mast cells [367]. In Wilms’ tumor, low expression of CCT4 can activate the ErbB pathway, increase the secretion of the inflammatory factors IL-6/IL-8, and create a pro-tumor inflammatory microenvironment [349]. In colorectal cancer, CCT6A can lead to CD8⁺ T-cell exhaustion by upregulating TUBA1B expression and is associated with reduced infiltration of CD4⁺ T cells, B cells, and dendritic cells in the tumor microenvironment [298, 368]. In pancreatic ductal adenocarcinoma, the key exosomal protein CCT6A derived from tumors can drive the polarization of tumor-associated macrophages toward the M2 phenotype ​​ [294]. Therefore, chaperonins can promote cancer cell immune escape by weakening anti-cancer immune responses and forming an immunosuppressive cancer microenvironment.

#### Chaperonins and cancer drug resistance

The expression level of chaperonins is directly correlated with chemosensitivity. In most cancers, high expression of chaperonins is closely associated with cancer drug resistance [233, 234, 242, 317, 369–375]. Chaperonins mediate cancer drug resistance through mechanisms such as modulating the cancer immune microenvironment, regulating mitochondrial function, affecting drug accumulation, stabilizing the cytoskeletal structure, and interfering with cell death pathways.

Regarding cancer immune microenvironment, the expression level of HSP60 is significantly greater in colon cancer patients who are insensitive to the mFOLFOX chemotherapy regimen than in those in the sensitive group. High HSP60 expression is positively correlated with the infiltration of immunosuppressive cells (e.g., DCs, FOXP3 + Tregs, and CD4 + T cells) and negatively correlated with the infiltration of cytotoxic cells (CD8 + T cells). HSP60 may contribute to drug resistance by recruiting immunosuppressive cells to weaken anti-cancer immune responses [369]. In HCC, the methylation of HSP60 by protein arginine methyltransferase 3 can inhibit the cGAS/STING innate immune pathway, impair T-cell-mediated anti-cancer immunity, and is consequently associated with resistance to immune checkpoint blockade therapy [360].

Regarding mitochondrial function, HSP60 can increase oxidative phosphorylation capacity by activating the mtUPRmt and upregulating β-catenin signaling, thereby providing energy for cancer cells and leading to drug resistance. Conversely, chemotherapy drugs can induce mitochondrial stress and ROS production. The upregulation of HSP60 helps to counteract chemotherapy-induced mitochondrial stress by stabilizing mitochondria and inhibiting ROS-dependent apoptosis, thus promoting drug resistance [253]. Therefore, inhibiting HSP60 can sensitize neuroendocrine prostate cancer cells to cisplatin and increase the sensitivity of glioblastoma to resveratrol [329, 359].

Regarding drug accumulation, chaperonins primarily affect the intracellular concentration of chemotherapeutic agents in cancer cells by promoting drug efflux and reducing drug influx. In AML, CCT1 upregulates the expression of drug efflux pumps (MRP1 and P-gp) by activating the PI3K/AKT signaling pathway, thereby reducing the intracellular accumulation of doxorubicin [376]. In ESCC, overexpression of CCT8 increases the expression of cytoskeletal proteins to maintain cell membrane integrity, which can reduce the influx of cisplatin [236].

Regarding cytoskeleton, chaperonins can directly affect the chemosensitivity of chemotherapeutic agents to microtubule polymerization/depolymerization by stabilizing the cytoskeleton. In acute lymphoblastic leukemia, CCT2 and CCT5 assist in the correct folding of actin and tubulin, stabilizing microtubules and counteracting the microtubule-depolymerizing effect of vincristine [377]. Similarly, loss of CCT3 leads to aberrant microtubule structure, impaired microtubule polymerization, and defective kinetochore‒microtubule attachment. Consequently, CCT3-deficient HCC cells become more sensitive to vincristine (which inhibits microtubule polymerization) but resistant to paclitaxel (which promotes microtubule polymerization) [378].

Regarding cell death pathways, chaperonins can resist chemotherapy-induced cell death by inhibiting apoptosis, autophagy and ferroptosis, thereby enhancing pro-survival signaling. In AML, CCT1 inhibits apoptosis and autophagy by activating the AKT/mTOR signaling pathway, increasing resistance to doxorubicin [371, 379]. In non-small cell lung cancer, CCT3 inhibits apoptosis by activating the JAK2/STAT3 pathway, increasing resistance to cisplatin [242]. The overexpression of CCT6A in colorectal cancer also inhibits apoptosis and autophagy, thereby increasing resistance to cisplatin [373]. In HCC, CCT3 binds to ACTN4, impeding the recycling of transferrin receptor protein 1 and inhibiting iron endocytosis, thereby interfering with sorafenib-induced ferroptosis and leading to drug resistance to sorafenib [380].

## Chaperonins as biomarkers and therapeutic targets

As reviewed in previous sections regarding the role of chaperonins in embryogenesis and human diseases, they often exhibit significant expression differences between pathological and physiological states, and are deeply involved in the pathogenesis of various diseases. Therefore, chaperonins possess considerable potential as clinical biomarkers and therapeutic targets. This chapter will focus on the research progress and current challenges of chaperonins as diagnostic and prognostic biomarkers, elaborate on therapeutic strategies and drug development for chaperonin-related diseases, and provide an outlook on future chaperonin-based therapies.

### Advances and challenges in chaperonins as diagnostic and prognostic biomarkers

Owing to aberrant expression levels and abnormal subcellular localization of chaperonins in various diseases, along with their detectability in accessible bodily fluids such as blood, urine, and exhaled breath condensate, chaperonins represent potential diagnostic and prognostic biomarkers [294, 322, 381]. In Alzheimer’s disease, coronary heart disease, and most cancers and autoimmune disorders, the mRNA, circRNA, protein, and autoantibody forms of chaperonins have demonstrated significant diagnostic and prognostic value [102–104, 137, 176, 177, 322, 382, 383]. However, because chaperonins participate in fundamental biological processes across virtually all organs and can be activated by diverse pathophysiological stresses (e.g., ischemia, hypoxia, inflammation, and cancer), chaperonins lack organ specificity and disease-pathology specificity.

Determining the expression level, subcellular localization, or protein activity of a single HSP60 or a single TRiC subunit has limitations for reliable disease diagnosis or prognosis prediction. Combining chaperonin detection with other indicators significantly enhances the clinical utility of chaperonin as a diagnostic and prognostic biomarker. The effectiveness of such combined diagnostic models has been validated for diseases, including non-obstructive azoospermia, HCC, and glioblastoma, demonstrating significant improvements in early diagnosis rates [59, 282, 290, 296, 382, 384–387]. Similarly, combined prognostic models have proven capable of predicting survival outcomes (e.g., OS, DFS) and clinical malignant outcomes (e.g., recurrence, metastasis) in patients with conditions such as HCC, gastric cancer, and lung adenocarcinoma [217, 258, 287, 289, 388–399]. Combined models can also predict treatment sensitivity to immunotherapy and chemotherapy in patients with breast cancer, lung cancer, and gastric cancer [255, 346, 400–404].

However, most current chaperonin-related diagnostic, prognostic, and drug resistance prediction models have been validated only within databases or relatively small, limited cohorts. Future clinical studies on chaperonin-based models should proactively incorporate multicenter, prospective external validation during the research design phase. Furthermore, collaboration with different medical centers is essential to establish consensus on core issues, including clinical data collection protocols, quality control standards for biomarker detection, and patient privacy protection. Such efforts are crucial to advancing chaperonin-related clinical research, ensuring that the findings are validated by real-world, multi-center, and multi-ethnic population data.

### Therapeutic strategies and pharmacological agents for chaperonin-related diseases

#### Expression-based therapeutic strategies for chaperonin-related diseases

Prior to selecting a treatment strategy for chaperonin-related diseases, it is paramount to determine the alterations in chaperonin expression within the disease, which includes changes at the mRNA, circRNA, lncRNA, protein, and autoantibody levels. From a quantitative perspective, alterations in chaperonin expression primarily involve upregulation or downregulation. Even changes in subcellular localization can be essentially viewed as a localized increase or decrease within the cell. Mechanistically, quantitative alterations can be classified as either primary or secondary. Primary alterations​ in chaperonins can act as the etiological cause of a disease by interfering with fundamental biological processes. Secondary alterations​ can be further subdivided into two categories: 1) compensatory alterations​ in response to pathophysiological stress, which serve a protective, chaperone function; and 2) decompensatory alterations​ resulting from persistent and severe pathophysiological stress. Only after clarifying the direction (up/down) and nature (primary/compensatory/decompensatory) of the chaperonin alteration can the appropriate therapeutic strategy be determined.​ For example, diseases driven by primary upregulation or decompensatory upregulation of a chaperonin would require an inhibitory therapeutic strategy. Conversely, in diseases where the upregulation is compensatory, it may be necessary to maintain elevated chaperonin expression levels. Therefore, based on the expression change driving the pathology, we categorize chaperonin-related diseases into those driven by chaperonin upregulation​ and those driven by chaperonin downregulation. The current landscape of pharmacological research for each category is shown in Table 1.

**Table 1:** Summary of chaperonin-based pharmacological agents

Drug	Category	Target	Disease	Reference
6-Shogaol	Inhibitor	HSP60	Non-small cell lung cancer	[252]
AuNR-P17	Inhibitor	HSP60	Triple negative breast cancer	[405]
AuTPP@TA-Fe NPs	Inhibitor	HSP60	Triple negative breast cancer	[406]
Cathelicidin antimicrobial peptides	Inhibitor	HSP60	Coxsackievirus B3	[199]
circCCNY	Inhibitor	HSP60	Hepatocellular carcinoma	[407]
Closantel	Inhibitor	HSP60	/	[408]
Epolactaene	Inhibitor	HSP60	/	[409]
ETB	Inhibitor	HSP60	/	[410]
IMH-BDP NPs	Inhibitor	HSP60	Cancer	[411]
KHS101	Inhibitor	HSP60	Glioblastoma Non-small cell lung cancer	[412, 413]
Mizoribine	Inhibitor	HSP60	/	[409]
Myrtucommulone A	Inhibitor	HSP60	Leukemia	[414]
o-carboranylphenoxyacetanilide	Inhibitor	HSP60	/	[409]
Rafoxanide	Inhibitor	HSP60	/	[408]
Suramin	Inhibitor	HSP60	African sleeping sickness	[408]
CT20p	Inhibitor	TRiC	Neuroblastoma	[216]
CT20p	Inhibitor	TRiC	Small cell lung cancer	[241]
Cytochalasin D	Inhibitor	TRiC	/	[415]
Cucurbitacin E	Inhibitor	TRiC	/	[415]
HSF1A	Inhibitor	TRiC	Pertussis	[205]
Latrunculin A	Inhibitor	TRiC	/	[415]
I-Trp	Inhibitor	CCT2	Triple negative breast cancer	[416]
Dihydroartemisinin	Inhibitor	CCT2	Glioblastoma	[417]
Cyclovirobuxine D	Inhibitor	CCT3	Colon cancer	[418]
Anticarin-β	Inhibitor	CCT4	Osteosarcoma	[341]
DCAF12	Inhibitor	CCT5	/	[419, 420]
PCV2 Cap	Inhibitor	CCT5	Porcine circovirus type 2	[421]
PARK2	Inhibitor	CCT5	Nasopharyngeal carcinoma	[219]
TRIM21	Inhibitor	CCT6A	Triple negative breast cancer	[422]
TRIM38	Inhibitor	CCT6A	Colon cancer	[423]
Curcumin	Inhibitor/Activator	HSP60	Neuroinflammation/Neuroblastoma	[424, 425]
DLN-BDNF	Activator	HSP60	Parkinson’s disease	[122]
Enzyme-digested phycocyanin	Activator	CCT4	Alzheimer’s disease	[107]
FH PRO for Men antioxidant capsules	Activator	TRiC	Idiopathic male infertility	[60]
Scutellarin	Activator	CCT6A	Myocardial ischemia‒reperfusion injury	[142]
ApiCCT1	Supplement	mHTT	Huntington’s disease	[117]
ApiCCT3	Supplement	Tau	Alzheimer’s disease	[108]
ApiCCT7	Supplement	Tau	Alzheimer’s disease	[108]
Human recombinant mitochondrial HSP60	Supplement	HSP60	/	[426, 427]
Human recombinant CCT	Supplement	TRiC	/	[428]
(HR + P)-EMV-lncCCT4-2	Carrier	CCT4	Myocardial ischemia‒reperfusion injury	[143]
shCCT2-UC-MSC-EVs	Carrier	CCT2	Liver ischemia‒reperfusion injury	[429]
Tat-CCT2	Carrier	CCT2	Cerebral and spinal cord ischemic injury	[430, 431]
McAb-A6-Au	Immune attack	CCT2	Acute lymphoblastic leukemia	[432, 433]
TETARs	Immune attack	CCT6A	Melanoma	[434, 435]
Galsomes	Immune tolerance	HSP60	Multiple myeloma	[338]
K409A pep	Immune tolerance	HSP60	Systemic lupus erythematosus	[179]
*Lactococcus lactis*-mbHSP65	Immune tolerance	HSP60	Atherosclerosis	[436]
mbHSP65	Immune tolerance	HSP60	Atherosclerosis	[437]
PgHSP60-derived peptide 14	Immune tolerance	HSP60	Atherosclerosis	[438]

#### Inhibitors of the HSP60 and TRiC chaperonins

For diseases driven by chaperonin upregulation, inhibitors targeting HSP60 and TRiC are under investigation. HSP60 inhibitors, such as KHS101, Myrtucommulone A, 6-Shogaol, Mizoribine, and Epolactaene, function by blocking ATP binding/hydrolysis or through covalent reactions with specific cysteine residues in HSP60, thereby inhibiting its function [252, 409, 412, 414]. TRiC inhibition​ can be achieved via non-subunit-specific inhibitors or subunit-specific inhibitors. Non-subunit-specific TRiC inhibitors​ include HSF1A, CT20p, Cytochalasin D, Latrunculin A, and Cucurbitacin E [205, 216, 241, 415]. Subunit-specific TRiC inhibitors​ target specific TRiC subunits for degradation and include I-Trp, Anticarin-β, DCAF12, PCV2 Cap, PARK2, TRIM21, and TRIM38 [219, 341, 416, 419–423].

#### Activator of the HSP60 and TRiC chaperonins

For diseases driven by chaperonin downregulation, therapeutic approaches aim to increase chaperonin levels, either indirectly or directly. Indirect upregulators​ include compounds such as Curcumin, DLN-BDNF, FHPRO for Men antioxidant capsules, Enzyme-digested phycocyanin, and Scutellarin, which can increase the expression of chaperonins [60, 107, 122, 142, 424]. Direct supplementation strategies​ involve administering the deficient chaperonin protein itself. Examples are Human recombinant mitochondrial HSP60, Human recombinant CCT, ApiCCT1, ApiCCT3, ApiCCT7, the Tat-CCT2 fusion protein, CCT2-UC-MSC-EVs, and (HR + P)-EMV-lncCCT4-2 [108, 117, 143, 426–428].

#### Competing endogenous RNA regulatory network

Regarding the bidirectional regulation of chaperonin expression levels, the competing endogenous RNA (ceRNA)-miRNA‒mRNA regulatory network serves as an important mechanism, as shown in Table 2. The ceRNAs (such as lncRNAs and circRNAs) within a cell share identical miRNA response elements, allowing them to competitively bind to the same miRNA. When a ceRNA successfully binds to a miRNA, it sequesters the miRNA, thereby relieving its inhibitory effect on the mRNAs of target genes and subsequently increasing the level of the protein it encodes [449, 457]. Consequently, by modulating the expression levels of specific ceRNAs or miRNAs, the bidirectional regulation of chaperonin protein levels can be achieved in preclinical models.

**Table 2:** Chaperonin-associated ceRNA regulatory network

ceRNA	miRNA	mRNA	Disease	Reference
/	miR-17	HSP60	Gastric lymphoma	[439]
/	miR-802-5p	HSP60	Cardiac insulin resistance	[440]
/	miR-382-5p	HSP60	Porcine reproductive and respiratory syndrome virus	[441]
/	miR-382-5p	HSP60	mtUPR in skeletal muscle	[442]
/	miR-29a	HSP60	Breast cancer	[443]
/	miR-1 miR-206	HSP60	Myocardial injury	[444]
/	miR-340-5p	CCT1	Acute myeloid leukemia	[445]
/	miR-24–3p miR-128–3p miR-149–5p	CCT3	Prostate cancer	[446]
/	miR-139-5p	CCT5	Hepatocellular carcinoma	[447]
/	miR-148a/152	CCT6A	Colon cancer	[448]
circCCT2	miR-671-5p	PRMT9	Hepatocellular carcinoma	[449]
circCCT3	miR-1287-5p	TEAD1	Hepatocellular carcinoma	[450]
circCCT3	miR-378a-3p	FLT1	Hepatocellular carcinoma	[451]
circCCT3	miR-135a-5p	PP2A	Bladder cancer	[452]
circCCT3	miR-223-3p	BRD4	Multiple myeloma	[336]
circCCT4	miR-338-3p	SOX4	Papillary thyroid carcinoma	[228]
circCCT2	/	PTBP1	Hepatoblastoma	[453]
circCCT8	/	/	Eosinophilic asthma	[454]
LncRNA CCT6A	/	/	Cerebral ischemia‒reperfusion injury	[455]
LncRNA LINC01503	/	HSP60	Colorectal cancer	[456]
LncRNA MALAT-1	/	CCT4	Lung adenocarcinoma	[245]
LncRNA CCT4-2	/	CCT4	Myocardial Ischemia‒reperfusion injury	[143]
LncRNA LINC00460	miR-503-5p miR-654-3p	CCT1	Hepatocellular carcinoma	[457]
LncRNA LINC01234	miR-30c-2-3p	CCT4	Breast cancer	[268]
LncRNA MALAT1	miR-101 miR-217	CCT4	Esophageal squamous cell carcinoma	[234]
LncRNA NEAT1	miR-152-3p	CCT6A	Glioma	[458]
LncRNA GAS5	miR-325-3p	CCT8	Posthemorrhagic hydrocephalus	[459]

#### Immune attack therapy and immune tolerance therapy

Beyond the straightforward strategies of supplementing or reducing chaperonin levels, the development of immune attack therapy and immune tolerance therapy based on the antigenicity of chaperonins has gained significant attention in cancers and autoimmune diseases, respectively. For diseases characterized by upregulated chaperonin expression, therapeutics have been developed that exploit chaperonins as antigenic targets. An example is the antibody‒drug conjugate (ADC) McAb-A6-Au, which consists of a cytotoxic drug linked to an antibody targeting a chaperonin [432, 433]. Additionally, T cells expressing two additional T-cell receptors (TETARs) can significantly increase the killing specificity of T cells. For example, personalized adoptive T-cell therapy, which simultaneously targets a common melanoma antigen (gp100) and the individual’s mutant antigen (CCT6A), has been developed for melanoma patients with CCT6A mutations [434, 435].

In autoimmune diseases triggered by aberrant chaperonin upregulation, immune tolerance vaccines containing chaperonin components aim to induce immune tolerance to the chaperonin antigen. Several HSP60 vaccine studies have shown promise. For example, oral HSP60 vaccines [460], intranasal HSP60 vaccines [461], multi-HSP60 epitope vaccines [462], and the injection of the complete tolerance-inducing adjuvant 8206 (which forms an active vaccine in situ with the endogenous pathogenic autoantigen HSP60) [463] have been shown in animal models to significantly reduce the size of atherosclerotic plaques [338, 436–438].

#### Pharmacological agents in clinical trials

Although most chaperonin-related therapies remain in the preclinical stage, simvastatin, DNA-hsp65, HspE7, DiaPep277, p336-351-CTB, CIGB-814 have advanced to the clinical trial stage, as shown in Table 3.

**Table 3:** Pharmacological agents in clinical trials

Drug	Stage	Clinical trial number	Disease	Reference
Simvastatin	Phase II	/	Dyslipidemia	[464]
DNA-hsp65	Phase I	/	Head and neck cancer	[465]
HspE7	Phase II	/	Respiratory papillomatosis patients	[466]
HspE7	Phase II	NCT00075569	Cervical intraepithelial neoplasia III	[467]
HspE7	Phase I/II	/	High-grade anal intraepithelial neoplasia	[468]
DiaPep277	Phase II	NCT01103284	Type 1 diabetes	[469]
DiaPep277	Phase II	/	Type 1 diabetes	[470]
p336-351-CTB	Phase I/II	/	Behcet’s uveitis	[471]
CIGB-258	Phase I	RPCEC00000238	Rheumatoid arthritis	[472]
CIGB-258	Phase II	RPCEC00000313	COVID-19	[473]

Simvastatin may indirectly reduce anti-HSP antibody levels by inhibiting the activation of NF-κB, suppressing T-cell activation, and downregulating CD40 expression. In a phase II clinical trial involving 102 dyslipidemic patients, treatment with simvastatin was associated with significant reductions in serum anti-HSP60 titers [464].

HSP65, which is present in both DNA-hsp65 (a plasmid DNA backbone carrying the specific *Mycobacterium* HSP65 gene) and HspE7 (a recombinant fusion protein comprising Hsp65 from *Mycobacterium bovis* BCG and the E7 protein from human papillomavirus type 16), also belongs to the HSP60 family. The roles of HSP65 in these drugs are to either 1) express the HSP65 protein to activate innate or adaptive immunity, or 2) serve as an adjuvant to enhance the immunogenicity of the HPV E7 antigen, thereby targeting cancer tissues or oncogenic proteins. In a phase I clinical trial involving 21 patients with advanced head and neck carcinoma, ultrasound-guided intratumoral injection of naked DNA-hsp65 plasmid induced partial tumor volume regression in 4 patients and stable disease in 1 patient [465]. In a phase II clinical trial involving 27 pediatric recurrent respiratory papillomatosis (RRP) patients, treatment with HspE7 improved the clinical course as it reduces the frequency of required surgeries [457]. In a phase II clinical trial involving 58 patients with cervical intraepithelial neoplasia III (CIN III), treatment with HspE7 promoted the regression of CIN III lesions, with 22.5% of patients achieving a pathologic complete response and 55% achieving a clinical partial response [467]. In a phase I/II clinical trial involving 15 HIV patients with high-grade anal intraepithelial neoplasia (HG-AIN), treatment with HspE7 resulted in the regression of lesions to AIN 1 or ASC-US in five patients [468].

DiaPep277, p336-351-CTB, and CIGB‑814 all contain peptide segments derived from HSP60. These peptides, either alone or in conjunction with adjuvants, can modulate T-cell responses by promoting a shift from a pro-inflammatory Th1 phenotype toward an anti-inflammatory Th2 or regulatory T-cell (Treg) phenotype. In a phase II clinical trial (NCT01103284) involving 35 adult patients with type 1 diabetes, DiaPep277 was demonstrated to modulate immune responses, effectively delay the loss of β-cell function, and improve glycemic control [469]. However, in another phase II clinical trial involving 30 pediatric patients with type 1 diabetes, DiaPep277 showed no beneficial effects in preserving β-cell function or improving metabolic control [470]. Unfortunately, the research results related to DiaPep277 in a phase III clinical trial (NCT00615264) were retracted due to allegations of academic misconduct [474]. In a phase I/II clinical trial involving 8 cases of Behcet’s uveitis, oral p336-351-CTB prevented the recurrence of uveitis [471]. In a phase I clinical trial (RPCEC00000238) involving 20 patients with moderate active rheumatoid arthritis, subcutaneous administration of CIGB‑814 significantly reduced IL‑17 and IFN‑γ levels, promoted clinical improvement and radiographic improvement [472]. In a phase II clinical trial (RPCEC00000313) involving 24 patients with severe and critical COVID‑19, intravenous administration of CIGB‑814 effectively controlled hyperinflammation and facilitated patient recovery [473].

### Future directions in chaperonin-based therapies

Although significant advancements have been made in both basic and pharmacological research related to chaperonins, the majority of findings remain at the preclinical stage. Enhancing the clinical translation rate of these research outcomes is a critical direction for future efforts. Based on the current landscape of drug development, we propose that the future development of chaperonin-based therapies should primarily focus primarily on the following six aspects.

1. Current research indicates that some combined models incorporating chaperonin can predict sensitivity to chemotherapy and immunotherapy, potentially guiding clinicians in drug selection and helping to avoid agents with a high probability of resistance upfront [255, 346, 400–404]. Future work should aim to expand the range of drugs these models predict and undergo external validation in larger patient cohorts.

2. Since current straightforward strategies for supplementing or reducing chaperonin levels lack organ or tissue specificity, such therapeutic interventions risk causing iatrogenic functional disorders or even diseases. Consequently, future research must focus on developing targeted delivery vehicles for chaperonin-related drugs, which will increase the precision of drug targeting, thereby minimizing therapeutic side effects.

3. The ceRNA regulatory mechanism is pivotal for modulating chaperonin expression levels and holds potential for bidirectional regulation. However, corresponding miRNA- and ceRNA-targeting therapeutics remain underdeveloped. The success of miRNA antisense oligonucleotide drugs such as CDR132L, which inhibits miR-132 for heart failure treatment and has passed phase I clinical trials, suggests a promising pathway [475]. Therefore, developing antisense oligonucleotides that target miRNAs or ceRNAs that regulate chaperonins represents a significant future direction for the treatment of chaperonin-related diseases.

4. The development of targeted drugs based on the antigenicity of chaperonins is an emerging trend. The specific antibody part of ADCs and TETARs is designed as a chaperonin protein-specific antibody, thereby enhancing the killing of chaperonin-expressing target cells [432–435]. This approach can be extended to develop ADC, CAR-T, and TETAR therapies carrying chaperonin-specific antibodies for a wider range of diseases driven by chaperonin upregulation.

5. Vaccines containing chaperonin components are key therapeutic strategies for chaperonin-induced autoimmune diseases. Preclinical research in atherosclerosis and clinical trials in type 1 diabetes, Behcet’s uveitis, rheumatoid arthritis, COVID-19 have shown that chaperonin vaccines can effectively modulate immune responses, control inflammation, and improve clinical outcomes [338, 436–438, 469, 471–473]. Building on these experiences—including antigen modification, adjuvant and carrier selection, and antigen‒adjuvant ratio optimization—future efforts should focus on developing chaperonin-based immune tolerance vaccines for treating a broader spectrum of autoimmune diseases.

6. While effective gene therapies are currently lacking for patients with chaperonin-related genetic disorders, approved therapies such as Exagamglogene autotemcel (Casgevy™) for transfusion-dependent β-thalassemia demonstrate the feasibility of using CRISPR/Cas9 to edit hematopoietic stem and progenitor cells ex vivo before reinfusing them to produce fetal hemoglobin [476]. Thus, developing gene therapy strategies using technologies such as CRISPR/Cas9 for chaperonin-related genetic disorders is a crucial future direction.

## Conclusion and outlook

Chaperonins​ specifically refer to HSPs with subunit/monomer molecular weights of approximately 60 kDa. In eukaryotes, Chaperonins primarily include HSP60 (Group I chaperonin) and TRiC (Group II chaperonin). Chaperonins are evolutionarily conserved; they facilitate ATP-dependent protein folding under physiological conditions and initiate stress responses under stressful conditions, thereby contributing to cellular homeostasis. Beyond these canonical chaperonin activities, chaperonins are involved in immune modulation, inflammation regulation, autophagy, the oxidative stress response, apoptosis control, and intercellular communication. Given the ubiquitous nature of the biological processes in which chaperonins participate and their body-wide distribution, chaperonin-related diseases are characterized by two distinctive features: systemic involvement across the body and manifestation throughout the entire lifespan. Aberrant chaperonin expression can significantly impact embryonic and organ development and contributes to the pathogenesis of genetic disorders, neurodegenerative disorders, cardiovascular diseases, inflammatory diseases, autoimmune diseases, infectious diseases, and neoplastic diseases. Consequently, chaperonins serve as crucial biomarkers for disease diagnosis and prognosis, as well as important therapeutic targets.

To enhance the clinical translation of chaperonin-related research (spanning basic, pharmacological, and clinical studies), we propose several future directions. These include: expanding the coverage of drug sensitivity prediction models to encompass more drugs and patient populations; improving the targeting specificity of chaperonin-related therapies; developing antisense oligonucleotides that target the ceRNA–miRNA–mRNA regulatory pathway; developing immune attack therapeutics carrying chaperonin-specific antibodies; developing immunosuppressive vaccines containing chaperonin components; exploring CRISPR–Cas9-based gene therapy strategies; and validating clinical research findings (e.g., diagnostic and prognostic models) using real-world, multi-center, multi-ethnic cohort data.

## Data Availability

Not applicable.

## Abbreviations

ADC: Antibody‒drug conjugate
AD: Alzheimer’s disease
ALS: Amyotrophic lateral sclerosis
AML: Acute myeloid leukemia
AS: Ankylosing spondylitis
ceRNA: Competing endogenous RNA
cGMP: Cyclic guanosine monophosphate
CP: Cleft palate
CSF: Cerebrospinal fluid
Ct: *Chlamydia trachomatis*
CVB3: Coxsackievirus B3
DAMP: Damage-associated molecular pattern
D2-MSNs: D2-type medium spiny neurons
EMVs: Endothelial microvesicles
ESCC: Esophageal squamous cell carcinoma
FMDV: Foot-and-Mouth Disease Virus
H/R: Hypoxia/reoxygenation
HAdV-C5: Human adenovirus type 5
HBV: Hepatitis B virus
HD: Huntington’s disease
HT: Hashimoto’s thyroiditis
HUVECs: Human umbilical vein endothelial cells
HPV: Human papillomavirus
Hp: *Helicobacter pylori*
IAV: Influenza A Virus
JEV: Japanese encephalitis virus
KD: Kawasaki disease
LAP: Listeria adhesion protein
LASV: Lassa virus
LN: Lupus nephritis
MCKD1: Medullary cystic kidney disease type 1
mHTT: Mutant Huntingtin protein
mtDNA: Mitochondrial DNA
mtUPR: Mitochondrial unfolded protein response
M. oralis: Methanobrevibacter oralis
nAAbs: Natural autoantibodies
PA1: First pharyngeal arch
PD: Parkinson’s disease
PolyQ: Polyglutamine
RA: Rheumatoid arthritis
RABV: Rabies virus
sGC: Soluble guanylyl cyclase
SLE: Systemic lupus erythematosus
TETARs: T cells expressing two additional T-cell receptors
TLR: Toll-like receptor
Tregs: Regulatory T cells
ZIKV: Zika virus
